# Delivery of therapeutic oligonucleotides in nanoscale

**DOI:** 10.1016/j.bioactmat.2021.05.038

**Published:** 2021-06-12

**Authors:** Lei Wu, Wenhui Zhou, Lihua Lin, Anhong Chen, Jing Feng, Xiangmeng Qu, Hongbo Zhang, Jun Yue

**Affiliations:** a School of Biomedical Engineering, Sun Yat-sen University, Guangzhou, 510006, Guangdong, China; b Pharmaceutical Sciences Laboratory and Turku Bioscience Centre, Åbo Akademi University, Turku, 20520, Finland; c Southern Medical University Affiliated Fengxian Hospital, Shanghai, 201499, China

**Keywords:** Therapeutic oligonucleotides, Nanoparticles, Anti-cancer, Targeted delivery, Clinical translation

## Abstract

Therapeutic oligonucleotides (TOs) represent one of the most promising drug candidates in the targeted cancer treatment due to their high specificity and capability of modulating cellular pathways that are not readily druggable. However, efficiently delivering of TOs to cancer cellular targets is still the biggest challenge in promoting their clinical translations. Emerging as a significant drug delivery vector, nanoparticles (NPs) can not only protect TOs from nuclease degradation and enhance their tumor accumulation, but also can improve the cell uptake efficiency of TOs as well as the following endosomal escape to increase the therapeutic index. Furthermore, targeted and on-demand drug release of TOs can also be approached to minimize the risk of toxicity towards normal tissues using stimuli-responsive NPs. In the past decades, remarkable progresses have been made on the TOs delivery based on various NPs with specific purposes. In this review, we will first give a brief introduction on the basis of TOs as well as the action mechanisms of several typical TOs, and then describe the obstacles that prevent the clinical translation of TOs, followed by a comprehensive overview of the recent progresses on TOs delivery based on several various types of nanocarriers containing lipid-based nanoparticles, polymeric nanoparticles, gold nanoparticles, porous nanoparticles, DNA/RNA nanoassembly, extracellular vesicles, and imaging-guided drug delivery nanoparticles.

## Highlights

• Therapeutic TOs are most promising drug candidates for cancer therapy.
• Successful delivery of TOs is the biggest challenge for clinic translation.
• Nanocarriers have revolutionized and greatly promote the delivery of TOs in vivo.
• LNPs and extracellular vesicles are most commonly developed for TOs delivery.
• New generation of delivery systems for TOs still need to be further developed.

## Graphical abstract

**Figure:**
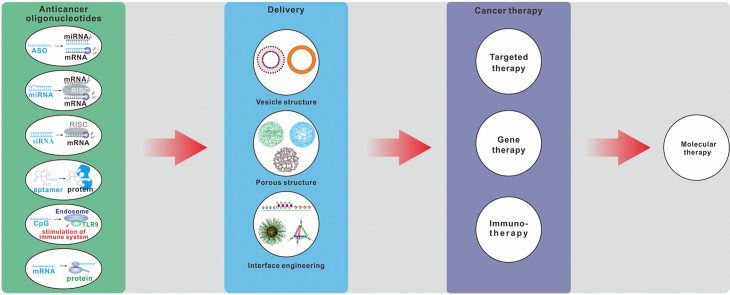

## 1 Introduction

Therapeutic oligonucleotides (TOs), short DNA or RNA oligomers, are an emerging category of drugs that can either interact with disease-associated genes via complementary Watson-Crick base pairing or recognize target proteins through the formation of three-dimensional secondary structures in a sequence-specific manner. With the rapid development of molecular biology tools, an extensive target gene profile responsible for the occurrence and development of specific diseases are being revealed, offering attractive opportunities for the development of oligonucleotide-mediated gene regulations for direct treatments of diseases or drug sensitizations. Comparing with the traditional small molecular drugs, TOs exhibit unique advantages such as the high selectivity to the target, possibility to target traditionally undruggable targets, capabilities to achieve personalized medicine, and negligible toxicity towards normal tissues [1]. TOs can be engineered without difficulty to prevent the flow of genetic information at all levels (replication, transcription, transformation, and recombination). Owing to their extraordinary controllability and adaptive structure, TOs is also commonly used for specific recognition and binding to cognate target molecules with high affinities to inhibit their functions, for example, through receptor-ligand interactions, or stimulating immune responses [2]. Since the first approval of antisense oligonucleotide drug, Fomivirsen, in the 1990s for the treatment of cytomegalovirus retinitis in individuals with AIDS [3], numerous TOs have now been approved from the FDA during the last two decades [[4], [5], [6]].

Gene based cancer therapy has been regarded as one of the most promising cancer treatments in the past several decades [7,8]. Some specific oligonucleotides, which have been regarded as the most efficient and natural gene regulation tools, such as antisense oligonucleotides (ASO), small interfering RNA (siRNA), microRNA (miRNA), messenger RNA (mRNA), aptamers, DNAzymes and immunoregulatory oligonucleotides have shown powerful theranostic activity against various cancers [[9], [10], [11], [12], [13], [14], [15]]. It should be noted however, deficient stability towards nuclease-mediated degradation and poor delivery efficiency to target organs of TOs seriously limited their wide-spread usage in clinic. As for cancer therapy, it is even more difficult for TOs to enter the tumor tissue and perform their anti-tumor activity because of the tumor microenvironment and physical barriers, which are totally different from the normal tissue [16]. To improve the stability of TOs, much efforts have been put into the chemical modifications of TOs on the internucleotide linkages [[17], [18], [19]], where a nonbridging oxygen is replaced by a sulphur atom. An in-depth discussion of these issues is outside the scope of this review and more information can be found elsewhere [20,21]. To overcome delivery obstacles, numerous strategies have been developed by designing versatile nanoparticles (NPs) that can load and transport TOs to the specific target in a programmable way [[22], [23], [24]]. Some of these NPs have positively-charged moieties that can form NPs-TOs assembly through electrostatic interactions, while others have the hydrophilic porous and hollow structures that can directly load TOs. By introducing specific functional groups to NPs that can sense the tumor microenvironment, programmable delivery and targeted release of TOs could be achieved. In the past decades, remarkable progresses have been made in the research of NPs-based delivery of TOs for cancer treatment [[25], [26], [27], [28], [29]].

**Scheme 1:**
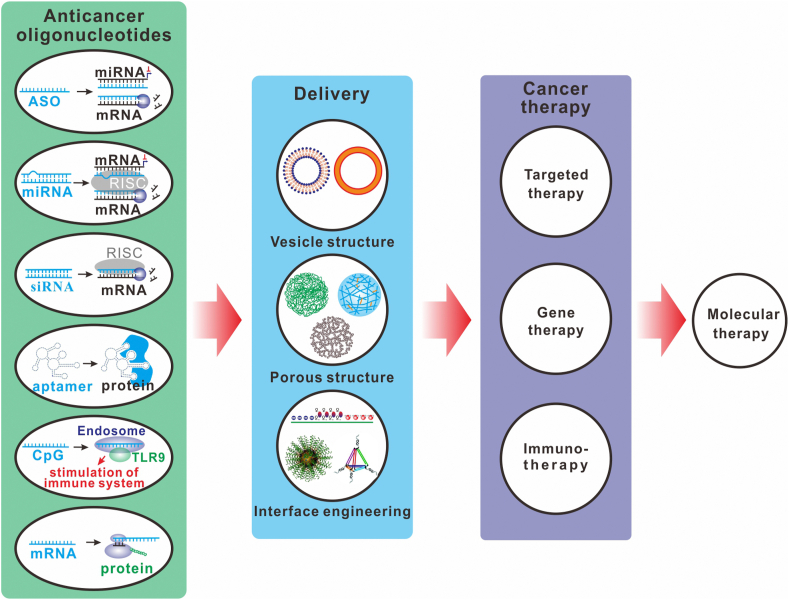
Nanocarrier-based systems for delivery of oligonucleotides for molecular therapy of cancers. Abbreviations: ASO, Antisense oligonucleotides; miRNA, microRNA; mRNA, messenger RNA; siRNA, small interfering RNA; RISC, RNA-induced silencing complex; TLR9, Toll-like receptors 9.

This review provides a comprehensive overview on the recent progress of nanocarrier-based systems for targeted delivery of TOs. Particularly we will focus on those TOs that have the therapeutic or modulatory activities specifically for cancer treatment (Scheme 1). Following a brief introduction of TO categorizations and the obstacles that prevent the clinical transformation of TOs, we will give a systematic overview on the TOs delivery based on the most widely used types of nanocarriers, including lipid-based nanoparticles, polymeric nanoparticles, gold nanoparticles, porous nanoparticles, DNA/RNA nanoassembly, extracellular vesicles, and imaging-guided drug delivery nanoparticles.

## 2 Classifications of the therapeutic oligonucleotides

Generally, therapeutic oligonucleotides are DNA or RNA strands that can be categorized into seven main groups: Antisense oligonucleotides, small interference RNA, microRNA, messenger RNA, DNAzymes, aptamers, and immunoregulatory oligonucleotides according to their different mechanisms in disease treatment.

***Antisense oligonucleotides (ASOs)*** ASOs are defined as a series of short single-stranded oligonucleotides that contain 16 to 20 nucleotides and are complementary to different classes of RNA [30]. Synthetic ASOs bind to RNAs via Watson-Crick base pairing and realize their capabilities in two main ways: RNA degradation or occupancy-only mechanisms [31]. RNA targeted degradation is one of the most classic ways of ASO to achieve gene inhibition with the help of ribonuclease H1 (RNase H1) and argonaute 2 endonuclease (Ago2) in the nucleus [32]. ASO usually contains a central 8 to 10 bases DNA gap called ‘gapmers’, which is the substrate of RNase H [33]. Occupancy-only mechanisms, also called space blocking mechanism, which will not lead to the targeted RNA degradation, but through change the RNA processing, such as cause exon skipping, or promote exon inclusion and also the subcellular localization of RNA to realize the gene silencing [30,34]. Antisense approach inhibits the expression of the certain disease-related gene is a promising therapeutic method because of the increased specificity and reduced side-effect toxicity [35]. ASOs are interesting in cancer therapy because their possibilities of decreasing the expression of oncogenic genes and targeting non-coding RNAs [36]. There are several ASOs based drugs currently in phase I clinical trials, such as STAT3 transcriptional inhibitor Danvatirsen (AZD9150) [37], TGF-β signal pathway inhibitor Trabedersen (OT-101) [38], the clusterin mRNA targeting second-generation ASO Custirsen (OGX-011) [39] and etc.

***Small interference RNA (siRNA) and microRNA (miRNA)*** can be regarded as two types of RNA interference (RNAi) that follow different pathways, which are forms of double stranded RNA (dsRNA)-mediated gene silencing at the transcriptional, posttranscriptional, and/or translational levels [40,41]. Both endogenous microRNAs and chemically synthesized small interference RNAs regulate gene expression with the help of Argonaute-containing RISC complexes [42,43]. miRNAs are noncoding RNAs with approximately 22 nucleotides, which can regulate protein-coding genes by partially binding to the 3′-untranslated region (3′UTR) of their mRNA targets. Generally, miRNAs inhibit target gene by translational repression, but they also increasing mRNA decay via deadenylation and decapping, resulting in degradation of these mRNAs or translational inhibition [44]. Since the recognition of miRNA and target mRNA can be realized by only a few consecutive complementary base paring, thus each miRNA can regulate the expression of multiple mRNAs and one of the target gene is regulated by multiple miRNAs at the same time. This may be a problem due to the lack of selectivity, while on the other hands, it provides a coordinate regulation of a gene clusters [21]. siRNA is a 22 to 25 basepair long RNA with a dinucleotide overhang at the 3’. siRNA consists of an antisense strand and a complementary sense strand, the antisense strand functionalize the activity of gene silencing, while the complementary sense strand are key factor that can help the intracellular transport of the antisense strand to RNA endonuclease Ago2 [31]. Therefore, RNA degradation is the most predominant manner that siRNAs realize their function. However, it's not like miRNAs, siRNAs cause cleavage but not translational suppression of target mRNA once bind to the targeted sequences [45,46]. The dysregulation of miRNAs expression at cellular level is always considered as one of the most important causes of tumorigenesis and cancer development [47]. This makes the supplementary of downregulated miRNA with miRNA mimics and inactivate the upregulated miRNA with antimiRNAs to be a common strategy for cancer therapy. The regulation of tumor related transcription factors and signaling pathway regulators with RNAi are also emerged as good way for cancer therapy. In 2018, the first RNAi based drug Onpattro (patisiran) [48] was approved by FDA in the application of Hereditary Transthyretin Amyloidosis treatment, and there are more than 20 siRNA, miRNA mimics and antimiRNAs in clinical trials [49,50].

***Messenger RNA (mRNA)*** mRNA, especially in vitro transcribed messenger RNA (IVT-mRNA) has also been considered as oligonucleotides in past several years. As a bridge between DNA and protein in eukaryotic cells holds great potential in vaccine development, protein replacement therapies and genetic diseases treatment, including cancers [51,52]. Compared with plasmid DNA and protein drugs, mRNA is much safer and more efficient. Firstly, mRNA functions outside the nucleus, which can avoid the potential insertion into the genome; secondly, the temporary bioactivity of mRNA makes the protein expression more controllable; thirdly, one mRNA molecule can generate multiple copies of a protein [53,54]. The immunogenicity, stability, translatability and also intracellular delivery of mRNA are the key factors for mRNA-based drug therapy [55,56]. With the modification of untranslated regions (UTRs), rare codons in protein-coding sequences and RNA bases, both enhanced mRNA stability, translatability and decreased immunostimulatory activity can be achieved [[57], [58], [59], [60], [61]]. And the in vitro transcribed messenger RNA (IVT-mRNA) has been developed as a new drug class for diverse therapeutic applications in the past several decades [51,62,63]. Whereas, methods and intracellular delivery system remain the major challenge for the broad application of mRNA therapeutics [55].

***DNAzymes*** DNAzymes are catalytically DNA molecules, mimicking a diverse range of enzymes. Some DNAzymes can bind to and cleave the mRNA of targeted genes, and some others can effectively catalyze RNA ligation or phosphorylation [64]. RNA-cleaving DNAzymes, which can cleave the sequence between any unpaired purine and pyrimidine of mRNA transcripts under physiological conditions [65], have gained more and more attention in the application of diagnosis and treatment owing to their perfect stability, modifiability and multiple selection of combined application [66]. Compared to ribozymes, DNAzymes are easier to synthesis, more stable and much cheaper in the application of disease treatment. In recent years, DNAzymes has been regarded as a good candidate for cancer therapy [66,67].

***Aptamers*** Aptamers, which are origin from Latin *aptus* (fit) and Greek *meros* (part), represent an emerging class of therapeutics. Aptamers, the folded three-dimensional structures of which can recognize various targets including small molecules, peptides, proteins and cells with high affinity are short single-stranded DNA or RNA molecules with 20–100 nucleotides [68,69]. Aptamers are referred to as “synthetic antibodies”, they bind to biological molecules via a very specific manner but not act through sequence-specific complementary paring. Comparing with antibodies however, aptamers have displayed more advantages in the design of moieties for the target recognition, e.g. they are much smaller and more stable than antibodies and can be chemically-modified in a defined and precise way, and most importantly they are non-immunogenic [70,71]. In contrast to ASOs or siRNAs, which must be delivered inside the cell, aptamers can target either extracellular or intracellular proteins [72]. Nowadays, aptamers have become an effective tool in the development of targeted delivery systems for both diagnostic and therapeutic purposes, and some specific aptamers, like AS1411, have attracted much attention in clinic for exploring their potential against a variety of cancer targets [72].

***Immunoregulatory oligonucleotides*** Except the above mentioned TOs, there is a special type of oligonucleotides, which act by modulating immunity for treating diseases in a targeted and specific manner. Immunoregulatory oligonucleotides either stimulate the immunity via binding to toll-like receptors (TLRs, e.g. TLR3, TLR4, TLR7/8, and TLR9) for innate immune recognition of pathogens, or antagonize TLRs to regulate aberrant immunity for treating autoimmune disorders [73,74]. Among these oligonucleotides, unmethylated cytosine-phosphate-guanine (CpG)-containing oligodeoxynucleotide, as TLR9 agonists, has become an effective and widely-used adjuvant in developing vaccines for cancer treatment [[75], [76], [77]].

## 3 Obstacles that prevent the clinical transformation of TOs for cancer therapy

Generally, the instability, reticuloendothelial systems (RES) clearance and renal excretion of nucleic acids in blood circulation are main challenges that limit the clinical transition of TOs. The main reason for TOs’ instability is the presence of abundant nuclease in the plasma. Obviously, considerable TOs will be degraded rapidly once being injected into the blood because the DNases and RNases widely exist in the plasma and tissues. Meanwhile, the mononuclear phagocytes of the RES can also eliminate TOs since the prominent role of RES in host is defense [85]. Because TOs are artificially synthesized in vitro, when they are administrated in vivo, they may face the risk of being labeled and recognized as ‘foreign invaders’ followed by uptake and clearance by RES, especially the Kupffer cells lined in the liver sinusoids and macrophages in the marginal zone of the spleen. In addition, molecules in the sizes of 3–6 nm or smaller are easily filtered by kidney [86]. A significantly large number of TOs, e.g. siRNA and uncharged morpholino antisense oligonucleotides are mainly in this size range and thus will be rapidly excreted out by the renal route [87]. Therefore, rational TOs delivery systems must be able to evade or at least to minimize the RES clearance and renal excretion. However, the tumor tissue appears to be smarter than the normal tissue in escaping from drug treatment, which brings another challenge for TOs in the application of cancer treatment.

***Limited drug penetration within tumor stroma*** In tumor tissues, the dense and aberrant extracellular matrix (ECM), a non-cellular meshwork consisting of structural proteins, glycoproteins, and proteoglycans [88], may significantly limit the diffusion of drugs to the deep site of tumors. This matter is particularly important for TO delivery because of their macromolecular nature. Compared to healthy tissues, where blood flow and lymphatic drainage are well-balanced [89], in tumor tissues, the lymphatic drainage is blocked while vasculature is extremely leaky, resulting in an elevated interstitial fluid pressure (IFP) [90]. The IFP gradually increased with the distance from the vessel, which hinders the homogeneous distribution of macromolecular drugs or nanomedicines throughout the whole tumor [[91], [92], [93], [94]]. Taken the above together, both the dense ECM and IFP limited the drug penetration within tumor stroma. Strategies to overcome this limitation include degradation of the tumor ECM proteins or downregulating their expression [95,96], opening epithelial junctions [97], vessel normalization [98], and remodeling the tumor stroma through targeting of tumor-associated macrophage [99,100]. Moreover, the tumor microenvironment conditions, such as lower pH that are considered as the consequence of the accumulation of acid metabolites and oxygen deficit at the tumor site might also the reason of failed TOs delivery [101].

***Cell***/***organelle membrane barriers*** To realize the anti-tumor activity, almost all of the different types of TOs must be internalized into the tumor cells. Among these TOs, only aptamers can bind to the target cell membrane proteins via their loop structures, followed by cell uptake through receptor-mediated endocytosis (RME). While for most of other unmodified TOs, the electrostatic repulsion between the negatively-charged TOs and as well as the negatively-charged cell membrane prevent TOs passing through the cell membrane [102]. By means of transport vectors or using nanocarriers, TOs can be internalized by cells through specific uptake mechanism [103,104]. However, the poor permeability of tumor cell plasma membrane caused by changes of membrane lipid organization and increased sphingolipids and cholesterol membrane content make it even more difficult for TOs to enter cells [105]. For example, ABC efflux pumps transporters such as the P-glycoprotein (P-gp), MRP1 and BCRP correlated to drug resistant have always been found to be upregulated in cancer cells [106]. The decreased influx transporters, such as solute carriers (SLC) on the membrane of tumor cells also make a great challenge for achieving considerable drug levels in cells in the similar way [107]. And it should also be noted that the internalized TOs are mostly trapped in the endo/lysosomal compartment, which means that additional strategies related to endo/lysosomal escape must be considered especially for TOs with the target located in the cytoplasm or even nucleus, e.g. antisense oligonucleotides, siRNA/miRNA, and mRNA. Over the past decades, plenty of strategies have been developed to overcome the problem of endo/lysosomal confinement. Proton absorbing materials, such as peptides with arginine, lysine and histidine [108], have a high buffering capacity between pH 7.2 and 5.0 and thus have been extensively utilized to design systems for lyso/endosomal escape. These materials act through the proposed mechanism of ‘proton sponge effect’ [109], which involves an extensive inflow of ions and water pouring into the microenvironment of endosomal thus consequently leads to the release of the entrapped components form the disruptive endosomal membrane. As for delivering TOs to the nucleus, the nucleus membrane may serve as another barrier to prevent TO entering the nucleus. Nuclear transport frequently occurs through nuclear pore complexes, nonetheless, TOs condensates are hard to pass through nuclear pore complexes (NPC) due to their large size [110]. For dividing cells, TOs can get in the nucleus during mitosis when the permeability barrier eliminates. Whereas, in non-dividing cells, TOs must pass through the nuclear membrane via the NPC, and the NPC only allows entry of molecules with the size up to 9 nm and less than 40 kDa through free diffusion [111]. Regarding to larger macromolecules, varieties of nuclear localization signal (NLS) peptides have been developed in an attempt to deliver TOs into the nucleus through NPC, which is an energy-dependent process [112]. NLSs are short clusters of amino acids which can bind to TOs either through covalent attachment or by noncovalent electrostatic interaction and mediate transnuclear transport through the NPC.

***Blood-brain barrier*** Delivering drugs to brain tumors is much more difficult because of the existence of the blood-brain barrier (BBB) [113], which consists of tightly linked endothelial cells supported by a network of pericytes and astrocytes and is impermeable to most of molecules as small as sucrose. Theoretically, macromolecular TOs cannot directly pass through the BBB to enter the brain parenchyma. Many attempts have been applied to deliver drugs across the BBB, such as using a hypertonic solution to temporarily open the tight junctions of the endothelial cells [114], using ultrasound to open BBB [115,116], and applying a vasodilator to enhance the vascular permeability [117]. However, the invasive disruption of BBB structures may bring up serious toxicity to brain parenchyma or undergo unrecoverable side effects. Therefore, designing new delivery systems with the capacity to across the BBB is still a big challenge, especially for noninvasively administrations of TOs for brain cancer therapy.

***The challenge of lyso/endosomal trap*** Because both of the naked TOs drugs and the TOs drugs carried with liposome delivery system or conjugate delivery system need to enter into the cell in the form of endocytosis for them to function [118]. Thus, the final destination of all delivered TOs are decided by lyso/endosomal. However, most of the TOs in the endosomes that are produced by cell uptaking will be trapped inside of the lysosomes after the fusing of endosomes and lysosomes following the acidification and maturation of the endosomes [119,120]. This leads to the final degradation of these trapped TOs because of the certain enzymes in the lysosomes. Currently, the adding of pH sensitive polymers, liposomes and CPP (Cell Caring Peptide) to the delivery system are widely used to increase the lyso/endosome escaping [121]. Whereas, the instability, high-sensitivity to the environment (especially the acidic microenvironment of tumor tissues) and also the lower targeting of pH-responsive delivery carriers remain the main problems that need to be solved. In addition, the discovery of GalNAC (N-Acetyl-d-galactosamine) delivery systems also gives a better solution for the delivery of TOs drugs targeting the liver [122]. But it cannot be used for the delivering to other target organs, because there are no similar expression level and recycling rate receptors as ASGPR has been found in other tissues. Therefore, other more efficient delivery systems, which can improve the delivery and utilization efficiency of TOs drugs by increasing endosomal escape are still needed to promote the clinical application of TOs drugs for cancer therapy [123].

***Off-target effects*** The off-target effects mainly consists of two reasons: one of the off-target effects is caused by RES clearance and renal excretion in blood circulation; the other reason is the off-target effect of some TOs. For example, the antisense strand of siRNA can not only mediate the silencing of homologous genes, but also cause the inhibition of some non-homologous genes through miRNA pathway. Meanwhile, the sense strand mediates the silencing of its homologous genes, causing the off-target effect mediated by the sense strand [124]. Otherwise, unmodified double-stranded RNA can also lead to the activation of innate immune response [125]. All of these off target effects will lead to toxic and side effects of TOs drugs and still need to be solved in the clinical application for cancer therapy.

In summary, the continuous improvement of non-viral nanoparticle carriers has provided a great opportunity for the application of TOs in cancer therapy, but the inefficiency of delivery carriers and lack of targeting are still the main problems restricting their clinical application.

## 4 Nanoparticles-based delivery of TOs

Generally, the loading of TOs to nanocarriers can be approached via physical or chemical methods. Negatively-charged TOs can either form complex with cationic nanocarriers via electrostatic interactions [126] or be loaded within the hollow structured nanocarrier with a hydrophilic inner cavity [127]. Besides, TOs can also directly bind to surface of metal nanoparticle through the facile Au–S chemistry [128,129]. Compared to the free TOs, TOs in nano formulations are much more advantageous: (1) NPs improve the stability of TOs. Except the chemical modifications of TO framework, using specific nanocarriers to form TO-NPs complex is an alternative way to improve the stability of TOs due to the shielding effect of nanoparticles. In addition, densely packing oligonucleotides on the NP surface has been reported as an effective way to prevent TOs from degradation by nuclease [130]. (2) NPs enhance the tumor accumulation of TOs. In the past 30 years, the enhanced permeability and retention (EPR) effect has become a central principle of passive targeting that drives the development of various nanomedicines for tumor-targeted drug delivery [131], though the importance and extent of EPR effect in human patients have been heavily debated in recent years [132,133]. Through relational design of NP size and shape, enhanced tumor accumulation of nanomedicines could be achieved [[134], [135], [136]]. (3) NPs improve cell uptake efficiency of TOs. Numerous studies have shown that NPs can enter cells as well as to translocate across the cell membranes through different cell uptake mechanisms depending on the structure of NPs [119,120]. Furthermore, through the surface functionalization of NPs with cancer-cell-targeting ligands [137], significant enhancement of cell uptake with high specificity can be achieved. (4) NPs can achieve targeted and on-demand drug release. To minimize the toxicity of nanomedicines to normal tissues, NPs can be designed with the capability to release their payloads only in the tumor tissues via responding to the tumor-specific microenvironments, such as hypoxia [138], acidic pH [139], irregular redox status [140], and so on. In addition, some smart NPs are capable of responding to external stimuli, like light [141,142], magnetic field [143], and ultrasound [144], during which process, controlled drug location and timing of administration, termed on-demand drug release could be realized.

Over the past few decades, striking progresses have been made in the advance of nanocarrier-based systems for targeted delivery of all types of DNA/RNA oligonucleotides. TOs can be loaded by nanocarriers through a variety of strategies, including physical encapsulations, electrostatic condensation, covalent binding, and Watson-Crick base-pairing. In the following sections, we will give a comprehensive overview on these progresses in terms of different formulations of nanocarriers: lipid (Section 4.1) and polymeric nanoparticles (Section 4.2), gold nanoparticles (Secion 4.3), porous nanomaterials (Section 4.4), DNA nanoassembly (Secion 4.5), extracellular vesicles (Secion 4.6), and imaging-guided drug delivery systems (Section 4.7).

### 4.1 Lipid-based nanoparticles

**Fig. 1:**
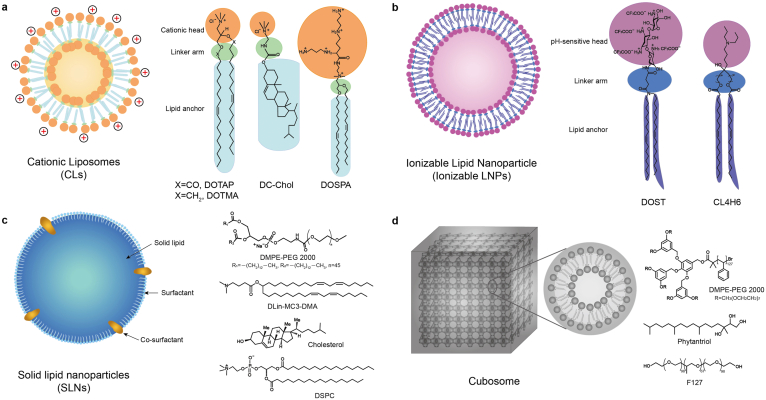
**Structural features of lipid-based nanoparticles**, including cationic liposomes **(a)**, ionizable lipid nanoparticles **(b)**, solid lipid nanoparticles **(c)**, and cubosomes **(d)**.

In the past decades, lipid-based nanoparticles (LNPs), composed of various lipid compositions and ratios with different structures, have shown great potential as delivery vehicles for TOs, containing antisense oligonucleotides, siRNA, and microRNA analogs [145]. To improve the stability and increase the delivery efficiency of LNPs, some helper lipids with specific geometry are usually added to LNPs [24], including the cone-shaped dioleoylphosphatidylethanolamine (DOPE, can promote endosomal release of TOs), cylindrical-shaped lipid phosphatidylcholine (can improve the stability of bilayer) and cholesterol (can improve the intracellular delivery of TOs and in vivo stability of LNPs). According to the structure and components of lipids, LNPs can be classified into four types: cationic liposomes, ionizable LNPs, solid LNPs, and cubosomes (Fig. 1).

#### 4.1.1 Cationic liposomes

Cationic liposomes (CLs) are vesicular structures mainly consisted of the positively charged lipids, which can interact with negatively charged DNA, forming cationic lipid/DNA complexes called lipoplexes [146,147]. Due to their high nucleic acids loading capacity, favorable interactions with cell membranes and versatile design of cationic lipids, which provides an efficient tool in screening liposomal formulations to achieve optimal gene therapy, CLs have gained much attention as gene delivery vectors for decades [145]. CLs are composed of a cationic hydrophilic head group, a hydrophobic lipid anchor group, and a linker arm bridged in between. Based on the structure of cationic head group, the cationic lipids can be classified into three major categories, (1) monovalent lipids, such as N (1-(2,3-dioleyloxy) propyl)-N,N,N-trimethylammonium chloride (DOTMA) [148] and 1,2-dioleyl-3-trimethylammonium-propane (DOTAP) [149], (2) multivalent lipids such as 2,3-dioleyloxy-N-(2(sperminecarboxaminino)ethyl)-N,N-dime-thyl-1-propanaminium trifluroacetate (DOSPA) and dioctadecylamidoglycylspermine (DOGS) [150], and (3) cationic lipid derivatives, such as 3β-(N-(N′,N'-dimethylaminoethane)-carbamoyl) cholesterol (DC-Chol) [151]. As for the linker arm, numerous studies have shown that the structure and orientation of linker bondshave a significant influence on the chemical stability and biodegradability of cationic head lipid, thus affecting their transfection efficiency and cytotoxicity [152,153]. Detail information based on how the structure of linker in cationic lipids influence the gene delivery efficacy of CLs can be found in a recent review [154].

CLs have demonstrated great potential in earlier studies in the targeted transportations of TOs [155,156]. To increase the DNA loading efficiency, massive cationic lipid are usually required in the formation of cationic liposomes [157]. However, high dose of cationic lipid administered in vivo might induce serious systematic toxicity. In addition, the strong interactions with serum proteins may lead to the rapid clearance of cationic liposomes from the circulation, decreasing the bioavailability [158].

Surface functionalization of nanoparticles (NPs) with PEG, or PEGylation, is one of the most extensively used strategies to enhance the stability and then prolong the fluid circulation of nanodrug delivery systems in vivo through reducing the opsonin adsorption on NPs [145]. However, it should be noted that PEGylation can prevent the NPs from interacting with the plasma membrane thus decrease their uptake efficiency by target cells. As for LNPs, inclusion of a PEGylating lipid can additionally hinder the fusion of LNPs with endosomal membrane upon internalization into cells, which seriously limits the delivery efficacy of cytosol-targeting therapeutics, e.g. siRNA [159].

On the one hand, the quaternary amine cationic lipid can supply high charge density to improve the loading of negatively charged oligonucleotides. On the other hand, the tertiary amine cationic lipid can provide a pH responsiveness for optimal delivery of oligonucleotides, therefore, Yung et al. developed a pH-sensitive carrier, QTsome, based on a combination of quaternary amine and tertiary amine cationic lipids [160]. Quaternary amine based cationic lipids are permanently charged, while tertiary amine based cationic lipids are mostly free of charge at neutral pH conditions and get fully ionized only at acidic pH, enhancing its ability to escape from the endosome compartment. QTsome loaded with TOs (AM-21) targeting to miR-21, a gene perticipated in multiple pathways regulating tumor progression and chemotherapy resistance, displayed high colloidal stability and good fusogenic activity in the endosome. Additionally, in vivo evaluations with tumor-bearing mice revealed that QTsome/AM-21 upregulated miR-21 target genes thus induced tumor regression, improved anti-tumor efficacy with chemo-gene therapy and prolonged survival.

#### 4.1.2 Ionizable LNPs

LNPs containing ionizable cationic lipids were developed to address the dilemma of CLs regarding their off-target effect and systemic toxicity induced by the highly charged cationic lipids despite their high drug loading efficiency. The functions of ionizable part are required to meet a number of tough challenges during the LNP-based TO delivery. Firstly, the lipoplexes is positively charged and adsorb negatively charged oligonucleotides forming the nanoparticle under the acidic conditions. Secondly, the ionizable part endows the LNPs with a distinct acid-dissociation constant (*pKa*) so that the surface of LNPs is close to neutral when they are at physiological condition. Thirdly, the lipid must assume a positive charge in the acidified endosome for purpose of interacting with naturally anionic phospholipids in the endosomal membrane and destabilize it [161]. Therefore two important factors should be taken into account when designing a proper ionizable lipoplexes: (1) the *pK*_*a*_ value of cationic head group, which is generally used to determine the pH condition for lipid gets protonation or deprotonation; (2) the capabilities of these ionizable lipids to induce a nonbilayer phase structure (hexagonal HII) upon protonation when they interact with endosomal membrane [145]. In recent years, ionizable LNPs have gained much notice owing to their negligible toxicity compared to CLs and versatility of structural variations of ionizable lipid, which provides an effective tool in screening optimal formulations for TO delivery [162]. Habrant et al. reported a series of novel ionizable carriers derivative from naturally occurring aminoglycoside tobramycin, which could form complex with various types of TOs, including mRNA, DNA, and siRNA [163]. After transfection potency evaluation of these carriers with structural changes at the level of linker and hydrophobic domain, the authors found that the lead molecules carrying biodegradable diester linkers exhibited the best transfection efficiency across all tested nucleic acids and cell types. Except the delivery of single formulation of TOs, ionizable LNPs can also be used to co-deliver multiple types of TOs. For example, Ball et al. co-formulated siRNA and mRNA that targeted to diseases-associated genes in a single lipidoid nanoparticle formulation, which consists of an ionizable amine-containing lipidoid, cholesterol, DSPC, DOPE, and PEG-lipid. They found that simultaneous delivery of siRNA and mRNA enhanced the efficacy of both drugs compared to their single counterparts, and with the addition of a negatively charged “helper polymer”, polystyrenesulfonate, the efficiency of the LNP drug delivery platforms have been dramatically improved [164].

#### 4.1.3 Solid LNP

Solid lipid nanoparticles (SLNs) typically contain a hydrophobic solid matrix core, which is usually comprised of biodegradable lipid components, and a surfactant shell designed to stabilize the SLNs. The lipids used to prepare SLN mainly comprise fatty acids (stearic acid), steroids (cholesterol), waxes (cetyl palmitate), monoglycerides, diglycerides and triglycerides. Different lipids and surfactants can affect the particle size, surface charge, long-term stability during storage, encapsulation efficiency and release profile [165]. Since 1990, SLNs have been developed as an alternative to nanoparticles, liposomes, and microparticles to deliver therapeutics because of their excellent biocompatibility and combinatorial advantages of polymeric nanoparticles, fat emulsions, and liposomes [166,167]. Furthermore, the preparation process without organic solvents makes the SLNs be of great potential for future clinical context.

A significant feature of SLNs is their ability to deliver hydrophilic and hydrophobic drugs depending on the preparation methods. Therefore, SLNs are promising carriers to co-deliver TOs and other types of therapeutics, such as small molecular drugs and biomacromolecules (e.g. polysaccharides, vaccine antigens) for combined therapy [[168], [169], [170]]. For example, Shi et al. developed a dual-drug-containing nano-vehicles for simultaneous delivering an endogenous microRNA (miR-34a) and paclitaxel (PTX) for synergistic cancer treatment. Results showed that the co-delivery system (miSLNs-34a/PTX) with an average size of approximately 220 nm could protect both miR-34a and PTX from degradation during the circulation. Furthermore, miSLNs-34a/PTX showed synergistic anticancer efficacy, where they displayed a much higher efficiency in inhibiting B16F10-bearing tumor growth compared to their single drug-loaded SLNs counterparts [171]. In another study, Kucukturkmen et al. utilized the high-pressure homogenization method to construct a cationic SLNs for co-delivering anti-miR-21 oligonucleotide, a TO targeting glioma-proliferation- and drug-resistance-associated gene (miR-21), and pemetrexed, a multi-targeted antifolate agent for treatment of brain tumors. A loading efficiency over 90% and the controlled release of pemetrexed were achieved, and cellular internalization of anti-miR-21 oligonucleotide/pemetrexed-coloaded SLNs by U87MG human glioblastoma cells was significantly higher and much more effective than that of free pemetrexed [172]. Although SLNs provides a feasibility to incorporate various oligonucleotides and lipophilic drugs with high payload, the preparation process of SLNs should be very carefully designed, considering that the stress and strain associated with the homogenization process may cause the fragile DNA/RNA, or other bioactive molecules degradation.

#### 4.1.4 Cubosomes

Cubosomes are colloidally stable cubic LNPs, which typically contain amphiphilic lipids with a unique internal bicontinuous cubic two-phase structure [173,174]. Since the first report by K. Larsson in 1989 [175], cubosomes have been regarded as promising nanovehicles for different routes of drug administration [176]. Cubosomes have a high internal and external surface area for loading both small molecular drugs and bioactive DNA/RNAs, owing to their continuously compartmentalized self-assemblies with hydrophilic and hydrophobic domains. Compared to conventional spherical LNPs, the unique curved structure of cubosomes provides an additional advantage for cytosolic delivery of siRNA, mRNA or any other TOs that needs to be escaped from endosomes.

**Fig. 2:**
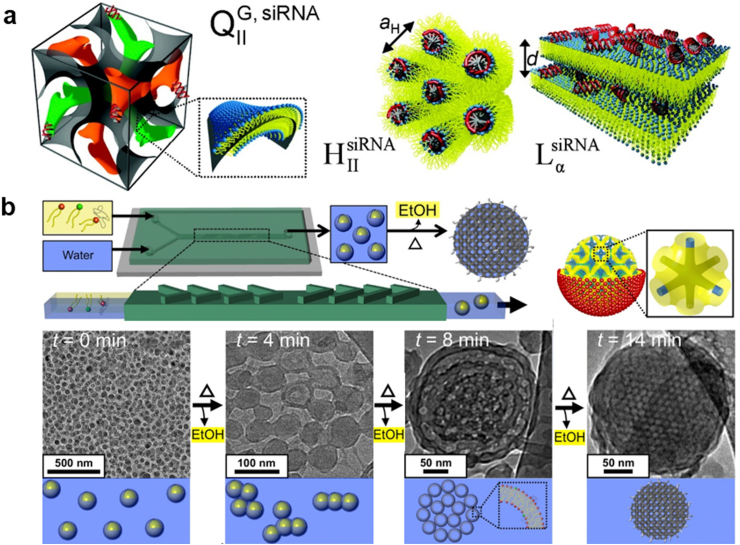
**Cubosomes-based NPs for siRNA delivery. (a)** Schematic depiction of the cubic (Q_II_^G, siRNA^) phase, the inverted hexagonal (H_II_^siRNA^) phase, and the lamellar (L_α_^siRNA^) phase of cationic liposomes/siRNA complexes, Reproduced with permission [177]. Copyright 2010, American Chemical Society; **(b)** Microfluidic synthesis of cubosomes and their formation mechanisms, Reproduced with permission [178]. Copyright 2018, American Chemical Society.

The cubosome-based TO-delivery system was first reported by Leal et al. where they developed an inverse gyroid bicontinuous cubic nanostructure composed of nonionic lipid glycerol monooleates (GMO) and small amounts of univalent cationic lipid DOTAP, which facilitated the incorporation of siRNA within its water channels [177]. Remarkable gene silencing was achieved due to the improved endosomal escape, which could be attributed to the formation of dynamical transient pores driven by the positive Gaussian modulus of the cubic phase membrane. In addition, the cubic lipid nanostructures did not show negative implications on cell viability and plasma membrane integrity because of their low charge densities. Interestingly, they found that the cubic (Q_II_^G, siRNA^) and the inverted hexagonal (H_II_^siRNA^) phase containing GMO exhibited higher total silencing and lower nonspecific silencing than the lamellar (L_α_^siRNA^) phase (Fig. 2a).

Ultrachilled sonication method was exclusively successful in the formation of cubosomes in earlier studies, while the obtained particle size was usually in micro scale and their size distributions were too broad, which were adverse for their clinical transitions. To overcome this obstacle, Kim et al. developed a microfluidic nano-manufacturing device to synthesize cubosomes based on GMO/DOTAP/GMO-PEG lipid mixture (Fig. 2b). The microfluidic device allows rapid mixing (0.6 s) of lipid/ethanol and water solutions, leading to the formation of 50 nm ethanol-in-water emulsion droplets that stabilized by the lipid layer. After the evaporation of ethanol, membranes self-organized into periodic bicontinuous cubic arrays to form cubosomes of approximately 200 nm with extremely narrow size distribution (PDI = 0.04). Moreover, when the amount of a steric stabilizer GMO-PEG was increased from 1 to 2 mol %, cubosomes with sizes as small as 75 nm were obtained without loss of internal structure. Finally, the small size cubosomes demonstrated a significant role in terms of delivering and eliciting specific gene knockdown in targeted cells [178].

### 4.2 Polymeric nanoparticles

**Fig. 3:**
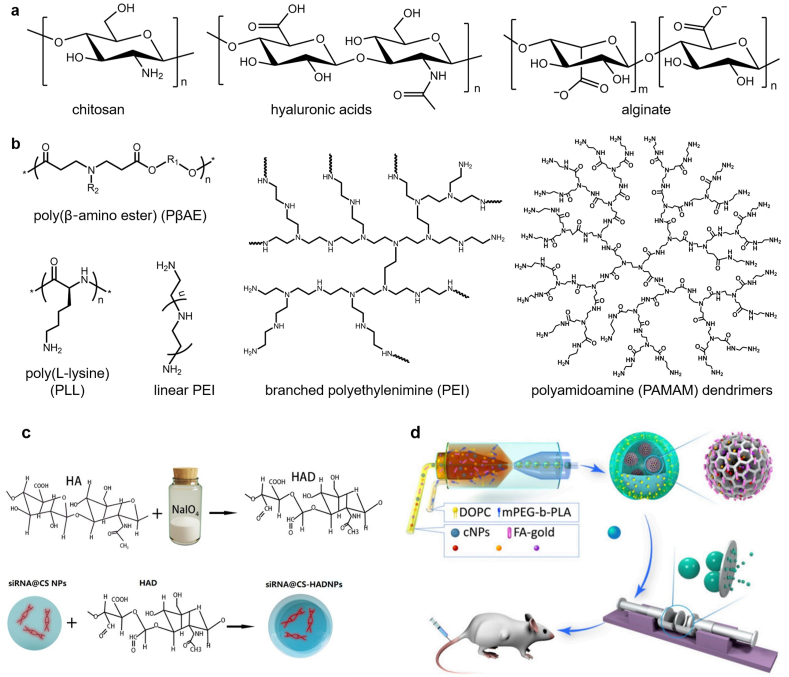
**Typical polymers used for TOs delivery.** (a) Natural–origin polymers-based NPs; (b) Synthetic polymers-based NPs; (c) Chitosan-hyaluronic acid dialdehyde nanoparticles (CS-HAD-NPs) for Bcl-2 siRNA delivery in the application of bladder cancer therapy [180]. Copyright 2021, Elsevier B.V.; (d) Schematic of core-shell structure based nanoparticles for drug delivery [181]. Copyright 2019, United States National Academy of Sciences.

Nanoparticles constructed from polymers, termed polymeric nanoparticles (PNPs), have been extensively explored in the anti-tumor delivery of various drug formulations due to their flexibility in tuning the physical-chemical properties for efficient drug loading and controllable release. The structural versatility endows PNPs with great opportunities in carrying TOs through multiple strategies, e.g. electro-adsorption by cationic polymer micelles, physical encapsulation within the cavity of polymer vesicles, or hybridization to PNPs constructed from amphiphilic DNA block copolymers via Watson-Crick base pairing [179], in pursuit of the optimal anti-tumor efficacy. Based on the polymer source of origin, PNPs can be divided into two categories: natural-origin polymers (Fig. 3a) based NPs and synthetic polymers (Fig. 3B) based NPs.

#### 4.2.1 Natural-origin polymers-based NPs

Natural-origin polymers, like chitosan, hyaluronic acids, and alginate (Fig. 3a) have received considerable interest for TO delivery due to their degradability, biocompatibility, and low cost [182]. Chitosan (CS) is a linear polysaccharide with free amino groups on the lateral chain, which can bind to TOs through electro-static interactions in a simple but efficient way. Chitosan NPs are biodegradable and have low immunogenicity, and their solubility as well as the biocompatibility can be largely improved through modifications of CSs with small molecules or polymers. For example, Sun et al. found that PEGylated CS had superior structural stability in the physical environment compared to unmodified CS nanoparticles [183]. PEG-CS NPs could effectively deliver siRNA to the targeting breast cancer cells and reduced the growth of xenograft tumors of 4T1 cells in vivo. In another study, Corbet et al. modified CS NPs with PEG through a simple non-covalent method, the surface of which was functionalized with the prototypical RGD peptidomimetic (RGDp) for tumor targeting [184]. The optimized RGDp/PEG-CS nanoformulations, with naphthyridine-containing RGDp randomly coupled to the PEG chain by clip photochemistry and the use of a lipophilic linker, have shown great capability in delivering siRNAs targeting to metabolic biomarkers of cancer cells: lactate and the glutamine transporters, leading to significant antitumor effects. Hyaluronic acid (HA) is an anionic, nonsulfated glycosaminoglycan that is the main component of the extracellular matrix (ECM). Due to their capability to target CD44-overexpressing tumor cells, HA has gained significant interest in the targeted anticancer therapy. In our recent work, targeting CD44 for bladder cancer treatment was achieved by delivering the Bcl-2 siRNA with self-crosslinkable chitosan-hyaluronic acid dialdehyde nanoparticles (Fig. 3c) [180]. It should be noted that HA cannot directly interact with oligonucleotides because of their electrostatic repulsion. However, this obstacle can be overcome by chemically modifying HA with specific groups for oligonucleotides conjugation or adding a third component of cationic polymers (polycations) to condense oligonucleotides [185]. In the latter situation, HA molecules usually locate on the surface of oligonucleotides-polycation complex, which endows the complex with the tumor targeting ability. Similar to HA, alginate, a natural polysaccharide consisted of α-d-mannuronic acid and β-l-guluronic acid, is another non-condensation oligonucleotides delivery system. In the presence of divalent cations (such as Ca^2+^, Ba^2+^), alginate nanogels with oligonucleotides-encapsulation capability can be obtained by precisely manipulating the diffusion of cations from a large outer reservoir into alginate solution [186]. Alternatively, alginate nanogel can be designed as a biocompatible shell decorated on the surface of polycation-DNAs complex, which can not only protect DNAs from nuclease degradation, but also enhance the transfection efficacy compared to non-decorated counterparts.

#### 4.2.2 Synthetic polymers-based NPs

Although natural polymers have shown excellent biocompatibility, their difficulties in controlling the differences between batches, their poor mechanical properties and limited processing capabilities mainly restrict their clinical transition. Comparing with natural polymers, synthetic polymers are recently being more attractive as carriers for TOs delivery owing to their reproducible properties on the basis of molecular weight, degradation, mechanical properties, and structural tunability to achieve efficient TOs loading and targeted release. The most frequently used synthetic polymers for TO delivery contain but are not limited to polyethylenimine (PEI), poly(l-lysine) (PLL), poly(β‐amino ester) (PβAE), polyamidoamine (PAMAM) dendrimers (Fig. 3b) [[187], [188], [189], [190], [191], [192], [193], [194], [195]]. One common feature of these polymers is that they all contain primary or tertiary amino groups that are protonable. After protonation, these polycations can form much small-sized polyion complexes (PICs) or polyplexes in aqueous media with anionic oligonucleotides through electrostatic interactions. The structural variability of polycations (e.g. the molecular weight, the charge density, the degree of branching) can be precisely tuned via controlling the polymerization conditions, which provides a feasibility for researchers to screen PNP formulations to achieve the best transfection efficiency. To efficiently condense TOs into nanoparticles, dense positive charged polycations with high molecular weight are generally required, which usually cause serious toxicity however. In contrast, low molecular weight polycations with low density of positive charges usually show poor efficacy in delivering TOs into cytosols. To overcome this dilemma, people used natural polyphenols, which have strong binding affinity with DNAs or RNAs via non-covalent ionic interactions, to achieve the condensation of nucleic acids by polycations with low molecular weight [196]. Effective TOs delivery with minimal toxicity was thus approached.

Using diblock copolymer PEG-*b*-PLL, Hayashi et al. investigated the influence of oligonucleotide rigidity on PIC formation by complexing the polymer with single-stranded RNA (ssRNA) or dsRNA. The PICs prepared from the flexible ssRNA showed a two-step assembly behavior: (1) formation of minimal charge-neutralized units (unit PICs); (2) formation of PIC micelles from unit PICs. In contrast, The PICs prepared from siRNA remained in the unit PICs stage throughout the measured range of concentrations, indicating that the rigidity of ionomers could be used as a pivotal structural parameter to stabilize the structure of the primary ion‐complex [187]. To overcome the instability of rigid siRNA-loaded micellar PICs, Naito et al. directly conjugated the siRNA to PEG-*b*-PLL through a reversible linker, tetravalent 3-fluorophenylboronic acid (FPBA), which can be replaced by the adenosine triphosphate (ATP) in a concentration‐dependent manner. At a low ATP concentration (<0.3 mM), the FPBA-crosslinked PIC micelles were quite stable, while at a higher ATP concentration (~3 mM, cytoplasmic condition), PIC micelles rapidly dissociated and released the siRNA payloads [192]. Except the strategies of loading TOs in the core of NPs, TOs can also be covalently immobilized to the surface of NPs without losing their potency. For instance, Chan et al. proposed a micellar NP based on the dual functionalized poly(d,l-lactide-*co*-2-methyl-2-carboxytrimethylene carbonate)-*graft*-poly(ethylene glycol), which was sequentially decorated with the targeting trastuzumab antibodies and siRNAs or antisense oligonucleotides (AOs) on the exterior PEG corona via the orthogonal Click reactions [197]. The targeted delivery systems are as effective as Lipofectamine in gene silencing without the associated potential toxicity of the latter. Moreover, the diblock copolymers can also be used as the shell structure of composite nanoparticles to achieve multiple drug delivery. In our previous work, a photothermal-responsive nanosized hybrid polymersome drug delivery system had been established for hydrophobic anticancer drugs delivery, and it can also be used for magnetic nanoparticles, DNA, or antibodies delivery to realized more specialized applications (Fig. 3d) [181].

As another widely developed biodegradable polymers with broadly adjustable structural diversity, poly(β-amino ester)s (PβAEs), mainly synthesized from a variety of diacrylates, amino-alcohols, and end-capping monomers by Michael addition reactions, have shown excellent efficiency in gene delivery [198]. A large number of PβAES libraries can be synthesized and screened with high throughput, generally with a simple synthesis procedure, to determine the optimal structures, resulting in efficient gene delivery to a wide range of cell types range of cell types [199]. To improve the stability of RNAi in serum, Dosta et al. synthesized hydrophobized versions of PβAEs using hexylamine, hexadecylamine, and cholesterol. The obtained polyplexes were stable against plasma proteins for more than 48 h and much higher transfection efficiency was achieved compared to non-hydrophobized PβAEs [194]. Besides the application in single gene delivery, PβAEs can also act as a potential carrier for combined delivery of TOs with proteins for synergistic cancer treatment. For example, Rui et al. prepared a novel class of hyperbranched PβAEs including both cationic and anionic charges through polymer end-capping with carboxylate ligands, and realized the gene editing function by co-delivery of gene-targeting short guide RNA (sgRNA) and Cas9 ribonucleoproteins (RNPs) [195]. The authors speculated that the introduction of carboxylate ligands can enhance the interactions between polymers and proteins, thus was beneficial for RNPs encapsulation. Furthermore, they found that the hydrophobicity of polymer end-group affected protein complexation, endocytosis of nanoparticles and escape from endosomes. Using optimized formulation of polyplexes, successful delivery of sgRNA/RNPs in vitro and in vivo and high levels of gene editing at relatively low RNP doses were achieved, highlighting the robustness and therapeutic potential of these nanocarriers.

Except the linear or hyper-branched polymers, tree-like dendritic polymers, termed dendrimers, are also promising carriers for TO delivery. In general, dendrimers mainly contain three distinct parts [200]: (1) a central core, where a dendrimer growth begins; (2) an inner shell, consisting of repetitive branching units (named as generations); and (3) an outer shell, comprised of numerous terminal functionalities attached to the surface. A representative example of dendrimer is polyamidoamine (PAMAM) dendrimer, which is consisted of interconnected ethylenediamine molecules through electrostatic interactions with oligonucleotides to form complexes [201]. It was found that the interactions of electrostatic oligodeoxynucleotide-dendrimer complexes were sensitive to pH as well as the ionic strength, and the maximal interaction occurred at low pH and ionic strength [202]. PAMAM dendrimers have inner cavities and peripheral functional groups, which that can be modified into physically embedding or covalently conjugate drugs with high curative effect [203], so as to become a promising platform for the co-delivery of genes and other drugs [188,204,205]. In addition, PAMAM dendrimers can be combined with other polymers to approach specific functionalities, such as sustained delivery of TO. For instance, Segovia et al. proposed a PAMAM dendrimer-based hydrogel system, embedded with siRNA loaded PβAE-NPs [206]. Local and continuous delivery of siRNA was achieved and the composite hydrogel displayed nearly twice the transfection efficiency in vitro compared to the most effective commercially transfection reagents. It should be noted however, PAMAM dendrimers with lower generations (G0-G4) usually have size smaller than 10 nm, which may face the risk of rapid renal clearance, while higher-generation dendrimers may produce higher cytotoxicity [207]. To decrease the cytotoxicity and improve the transfection efficiency, strategies focusing on the surface engineering of PAMAM dendrimers with specific moieties, e.g. lipids, peptides, amino acids, polymers, have been extensively explored [208,209]. Alternatively, interests have been put into the composite systems, where small sized PAMAM dendrimers were used as one of the building components in couple with other NP formulations. The fabricated composite delivery systems could sense the tumor microenvironment and programmable drug delivery and deep tumor penetration could be achieved [190,193,210,211].

### 4.3 Gold nanoparticles

Gold nanoparticles (AuNPs) is another broadly developed nanocarrier for targeted delivery of TOs in a variety of cancer cell lines due to their (1) precise control over sizes in the range of 2–200 nm; (2) facile surface functionalization with any kinds of TOs; (3) tunable localized surface plasmon resonance (LSPR) in a broad wavelength spectrum from visible to near IR region; (4) ease of qualitative and quantitative analysis in the complicated biological environment.

#### 4.3.1 Spherical gold nanoparticles

**Fig. 4:**
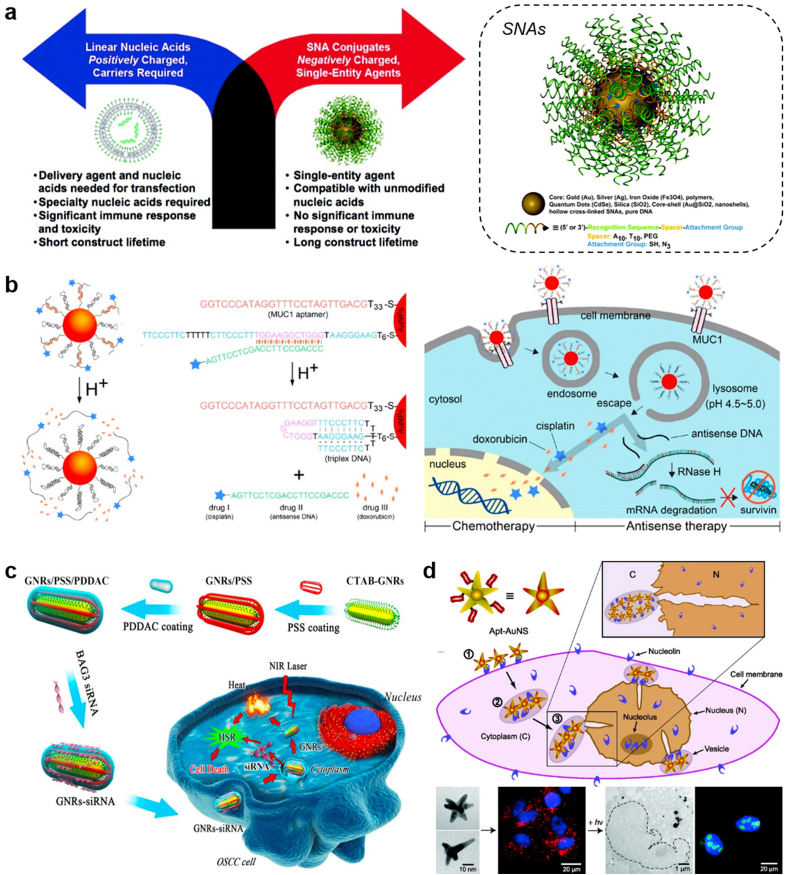
**Gold nanoparticle-based platforms for TOs delivery. (a)** Spherical nucleic acids offer a different paradigm for gene regulation. Reproduced with permission [215], Copyright 2012, American Chemical Society; **(b)** Multidrug-loaded nanoswitch and its intracellular pH-responsive multidrug delivery and release. Reproduced with permission [233], Copyright 2019, American Chemical Society; **(c)** BAG3 siRNA loaded gold nanorods to improve the PTT efficiency via silencing the heat-shock response. Reproduced with permission [238], Copyright 2016, Elsevier Ltd.; **(d)** Nucleolin-mediated, active trafficking of AS1411-conjugated gold nanostars to the cancer cell nucleus. Reproduced with permission [241], Copyright 2012, American Chemical Society.

In 1996, the Mirkin group constructed a spherical form of nucleic acids using the AuNPs template, termed as spherical nucleic acids (SNAs) [212]. The densely and highly oriented packing of DNAs on the surface of AuNPs sterically prevent DNA degradation by nucleases [130], while the hybridization capability of ssDNA on SNAs to its complimentary sequence did not decline. Moreover, SNAs can enter cells in high numbers via scavenger receptor mediated endocytosis without the assistance of transfection reagents [213,214], offering a new paradigm for gene regulation (Fig. 4a) that is different with traditional strategies [215], where negatively charged nucleic acids are always need to be pre-complexed with positively-charged carriers to enter cells. Up to now, dozens of SNAs have been developed and evaluated by Mirkin group for their potential in rapid diagnostics of cancers, as well as for various diseases that are difficult to address with traditional therapeutic strategies [129,216]. For example, in one earlier study, they fabricated the RNAi-based SNA targeting oncogenes (*Bcl2L12)* which is traditionally untargetable by small molecules or antibodies in glioblastoma multiforme (GBM) pathogenesis [217]. Upon intracranial or intravenous administration, *Bcl2L12*-SNAs could cross the blood-brain barrier (BBB), penetrate the glioma, and promote apoptosis of glioma cells by enhancing caspase and p53 activities. In another study, they fabricated immunomolulatory oligonucleotides-based SNAs to stimulate (IS–SNAs) or regulate (IR-SNAs) immunity by engaging TLRs [218]. Compared with the free oligonucleotides, IS-SNAs exhibited an 80-fold increase in responses to model antigens, which IR-SNAs have reduced fibrosis score in mice with nonalcoholic steatohepatitis by 30%. Recently, they expanded the type of SNAs by choosing different core materials, including liposome [[219], [220], [221]], polymeric micelles [222,223], proteins [224], and metal organic framework [225,226]. Such well-defined three-dimensional SNAs exhibit distinctive properties that apart from those of both the nanoparticles and nucleic acids form they derive, such as minimal immunogenicity [227,228], negligible cytotoxicity and undetectable off-target effects [229].

In addition to the use as the single formation for cancer treatment, TOs-loaded Au nanospheres can also be combined with chemotherapeutics to achieve enhanced anticancer therapy through synergistic effect between TOs and chemotherapeutics [[230], [231], [232]]. In an earlier study, Kim and his coworkers constructed a pH-responsive gold nanocluster formed from 13 nm of spherical Au NPs grafted with two types of oligodeoxynucleotides (ODNs), bcl-2 antisense ODN and i-motif binding ODN (iBO, a four-stranded DNA secondary structure that partially complementary to the i-motif, which usually forms in acidic conditions with cytosine-rich sequences) [231]. At neutral pH, addition of the i-motifs as the linker strands clumped Au NPs together through the partial hybridization of i-motif and iBO, and this duplex formation facilitate the loading of anticancer drug Dox by intercalation. After being internalized to the endosomes, however, the cluster could rapidly disassemble because of the formation of i-motif structure of the linker strands within acidic environment, which resulted in exposure of antisense ODNs to mRNA and enhanced Dox release for cancer cell killing. Compared with the single AuNPs as the drug carrier, size-tunable gold nanoclusters designed in this study provided more opportunities for both TOs and chemotherapeutics loading and stimuli-responsive drug release capacities [232].

Recently, Chen et al. developed a DNA-based nanodevice capable of releasing antisense DNA and chemotherapeutics responding to a pH range of approximately 5.0–7.0 [233]. The nanodevice was fabricated from AuNPs functionalized with the sequence-specific DNA complex, consisting of an anti-MUC1 aptamer (function as the targeting ligand), a conformation switchable DNA sequence in responsive to pH change, and antisense DNA (asDNA) targeting the cancer-associated survivin mRNA (Fig. 4b). The model drug Dox could be effectively loaded in the nanodevice via intercalation in the double strand region of the DNA complex. In lysosomes (pH ~4.5–5.0), the switchable DNA strand rapidly changed the conformation from linear (pH 7.4) to triplex, leading to the release of Dox and asDNA for synergistic cancer cell killing. Finally, the nanoswitch displayed an efficient gene silencing and a significant tumor-growth inhibition in the tumor-bearing mouse model.

#### 4.3.2 Non-spherical gold nanoparticles

Compared to sphereical AuNPs, non-spherical AuNPs (e.g. nanorods, nanostars, nanocubes), also called anisotropic AuNPs, have gained more interest in recent years in targeted delivery of TOs for anticancer therapy. Firstly, the anisotropic character of non-spherical AuNPs allows the LSPR peak of AuNPs shift from the visible region to the near infrared region (NIR), which enables researchers design NIR-responsive TO delivery systems to achieve deep tumor penetration and therefore enhance therapeutic index of TOs; Secondly, because of their optical response, anisotropic AuNPs show excellent optical signal enhancement, which can be used in the design of sensors, in addition to acting as the drug carrier, to probe the delivery as well as therapeutic efficiency of TOs in real time; Thirdly, due to the electron-phonon interactions, the NIR radiation absorbed by the anisotropic AuNPs surface can be rapidly converted into heat, which have been widely used in NIR-based photothermal therapy or photothermal/TOs combination therapy; Fourthly, numerous studies have shown that some specific anisotropic AuNPs have higher cellular uptake efficiency compared to spherical AuNPs, and the intracellular transportation of TO payloads with higher anticancer therapeutic index can also be realized by the anisotropic AuNPs.

The precise tunability in terms of sizes and aspect ratios of gold nanorods (AuNRs) provides many attractive optical properties for sensitive bioimaging and remote-controlled TOs delivery [234]. AuNRs are usually synthesized by seed-mediated reduction of tetrachloroauric acid in the presence of cetyltrimethylammonium bromide (CTAB) [235]. It is not easy to attach thiolated DNA on AuNRs is not easy because CTAB can be tightly adsorbed on AuNRs surface, which prevents the formation of an Au–S bond between thiolated DNA and AuNRs. Therefore, to further expand the potential of AuNRs in TOs delivery, the efficient and facile DNA-functionalization strategies must be developed. In pursuit of this purpose, Li et al. used mPEG-SH to replace CTAB on the surface of AuNRs and Tween 20 to assist the replacement process and stabilize AuNRs, and this complete functionalization could be finished within 1 h [236]. In another study, Wang et al. presented a potent strategy for loading siRNA duplexes onto AuNRs based on the dithiocarbamate chemistry [237]. They systematically evaluated the bioactivity of AuNRs–siRNA complexes against eGFP-producing ovarian cancer cells (SKOV-3). Efficient knockdown was achieved by on-demand release of DTC-anchored siRNA upon femtosecond-pulsed laser irradiation. Interestingly, using the same AuNRs, the knockdown activity of DTC-anchored siRNA was much higher than that of thiol-anchored siRNA. Non-invasive NIR photothermal therapy (PTT) based on AuNRs has become a promising tool in cancer treatment. To minimize the hyperthermia-induced collateral damage to normal tissues, a mild laser power or agent to produce moderate heat is suggested. However, mild PTT can trigger the heat shock response (HSR) in cancer cells, upregulating the expression of heat shock proteins and inhibiting the pathways of cell apoptosis. To overcome this dilemma, Wang et al. loaded siRNA targeting BAG3 (an HSR-associated gene) on AuNRs (Fig. 4c), which can effectively deliver the siRNA into cancer cells in silencing the heat-shock response [238]. The in vivo studies demonstrated that AuNRs-siRNA could effectively reduce the HSR in cancer cells and were sensitive to PTT following enhanced cell apoptosis under moderate laser irradiation.

The gold nanostars (AuNS) with a multi-branched morphology is another type of anisotropic AuNPs that have been widely developed in the targeted delivery of anticancer TOs. Comparing with gold nanorods, the synthesis of AuNS is much easier and greener: they can be synthesized by the ‘one-port, seedless’ reduction of Au^3+^ in biocompatible Good's buffer (e.g. HEPES, MOPS, EPPS) and do not need the toxic surfactant for stabilization, which make the post-synthesis loading of TOs more efficient [239]. From this aspect, AuNS seems more suitable than AuNRs when developed as the TO vehicle. In the past ten years, the Odom group developed a series of AuNS-based system for targeted delivery of TOs [[240], [241], [242], [243], [244], [245], [246]]. In one earlier study, they fabricated the AS1411 (a nucleolin specific aptamer)-conjugated AuNS and directly observed that the nanoconstructs could be locatedin the nucleus and cause major changes in nuclear phenotype through nuclear envelope invaginations (Fig. 4d), leading to the apoptosis of cancer cells [241]. Later on, they found that AS1411@AuNS exhibited notable anticancer effects in a group of 12 cancer cell lines [240]. In another work, they fabricated lysosomal targeting nanoconstructs composed of anti-HER2 aptamer (human epidermal growth factor receptor 2, HApt) grafted onto AuNS surface [243]. Within lysosomes, HER2 could be degraded by enzymes under acidic pH condition, leading to apoptosis of cancer cells. This work demonstrates that by targeting lysosomes and utilizing biomarkers for lysosomal degradation, the known shortcomings of therapeutics in nanoscale-the inactivation of TOs due to the entrapment of nanoconstructs in endo/lysosomes-can be overcome. Recently, the same group investigated how the surface curvature of AuNPs decorated with immunostimulatory oligonucleotides CpG influence the immune activation effects of CpG-AuNPs [244]. Interestingly, they found that the mixed-curvature constructs (nanostars) produce a relatively high percentage of hollow endosomes and a higher immune cell response than constant-curvature constructs (nanospheres) constructs. This work highlights that the local organizations of CpG inside endosomes, for pursuing optimal immune responses and intracellular delivery, can be controlled by using the anisotropic AuNS. Recently, our group found that the HApt-conjugated AuNS could also act as the promising carrier for incorporation of small molecular drugs (e.g. anticancer doxorubicin, Dox) to achieve on-demand drug delivery and enhanced toxicity against cancer cells [247].

Ultrasmall (~2 nm) AuNPs can directly deliver oligonucleotides to the nucleus without any additional nuclear-targeting functional ligands, which can interfere with the transcription process of related genes, thereby affecting the life activities of cells, and thus showing great anti-tumor potential. However, their low degree of net cellular uptake because of exocytosis and rapid clearance from the body limited the in vivo therapeutic efficacy [248,249]. To address these problems, Huo et al. designed a sunflower-like gold-DNA nanostructure (~200 nm), which was assembled from 2 nm AuNPs modified by the complementary silent sequence of the c-myc oncogene [250]. The nanosunflowers had a strong NIR absorption ability, and could decompose and release ultrasmall Au NPs under NIR irradiation. Increased cellular uptake, tunable gene silencing, and controlled tumor inhibition were successfully achieved by synergistically regulating the pre-incubation time and the NIR irradiation time point, the cell uptake was obviously increased while the adjustable gene silencing and a significant inhibitory effect on tumors were achieved.

Except the covalent immobilization strategies, TOs can also physically absorb to the surface of AuNPs pre-modified with the cationic polymers through electrostatic interactions. For example, Lei et al. developed a siRNA-loaded gold nanocluster system (GNC-siRNA complex) to target the interactions between tumor cells andneuron against cancer via depleting the nerve growth factor (NGF) [251], a kind of neurotrophic factor that actively promote the growth of neurites and stimulate neurogenesis, contributing to the survival, proliferation, invasion, and metastasis of tumors [[252], [253], [254], [255]]. GNCs that constituted by a straight but effective method endowed the complex with a high loading capacity of siRNA which significantly reduced NGF expression as well as the neurite sprouting, and eventually inhibited the tumor growth by activating a variety of downstream protein kinases. This study showed an attractive therapeutic direction to cancer treatment. In another study, Yi and coworkers reported a sub-50nm nanoassembly for target delivery of siRNA to cancer stem-like cells (CSCs). The nanostructure was prepared via a two-step assembling process, where lipoic acid-modified, glucose-installed poly(ethylene glycol)-*block*-poly(l-lysine) was first associated with siRNA via electrostatic interaction to form an unimer polyion complex (uPIC), which was further decorated on 20-nm Au NPs through Au–S bonding. As the core, Au NPs make it easier and more precise to obtain sub-50nm nanostructures compare to other formulations of nanocarriers for further efficient permeation of tumor tissues. The nanoassembly elicited significantly gene silencing effect and successfully suppressed the growth of the CSC-rich orthotopic breast tumor [256].

### 4.4 Porous nanomaterials

Porous nanomaterials are composed of a solid framework with porous structures and large surface area, which allow the high-efficiency encapsulation of drug molecules within the pores and facile modification of interest functional groups on the surface. The rapid development of synthetic methodologies of porous nanomaterials in recent years has largely expended the delivery applicability of drug moieties, either hydrophobic or hydrophilic, from traditional small molecules to more potent macromolecular drugs, like proteins and nucleic acids. Moreover, the pores inside SiNPs allow high-efficiently loading both oligonucleotides and compounds for combined cancer treatments. Numerous studies have indicated that through rational design of the pore size and morphology, porous nanomaterials can a promising vehicle in carrying TOs for the targeted cancer therapy. Generally, the widely developed porous nanomaterials for TOs delivery can be classified into three types: porous silicon nanoparticles, mesoporous silica nanoparticles and mental-organic framework. Compared to other types of nanoparticles, porous nanomaterials have uniquely mesoporous structures, high drug-loading capacity, high active surface area and potential for the development of gated supports for on‐demand delivery applications, and other unique characteristics that make them great potential alternatives for cancer treatment applications [[257], [258], [259]].

#### 4.4.1 Porous silicon nanoparticles (pSiNPs)

**Fig. 5:**
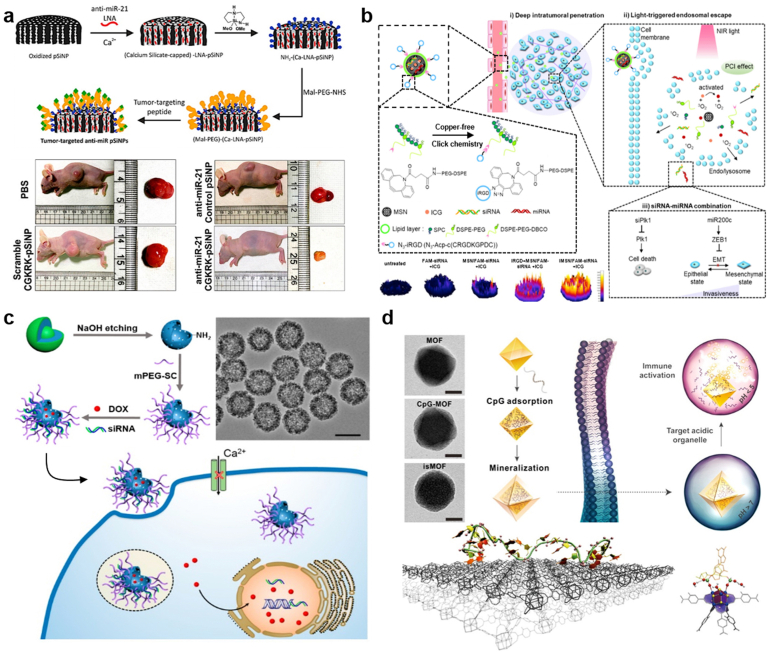
**Targeted delivery of TOs based on porous nanoparticles. (a)** Preparation of tumor-targeted anti-miR porous silicon nanoparticles (pSiNPs). Reproduced with permission [260], Copyright 2019, American Chemical Society; **(b)** NIR-Triggered RNA co-delivery by tumor-penetrating mesoporous silica nanoparticles (iMSNs) for siPlk1/miR-200c combination therapy. Reproduced with permission [268], Copyright 2020, American Chemical Society; **(c)** Fabrication of Dox/siRNA-loaded mesoporous silica nanoparticles to regulate Ca^2+^ signaling for drug-resistant breast cancer therapy. Reproduced with permission [271], Copyright 2019, American Chemical Society; **(d)** Coordination-based method for preparing immunostimulatory DNA–MOFs. Reproduced with permission [290], Copyright 2017, American Chemical Society.

Porous silicon nanoparticles (pSiNPs) can be synthesized either through physical methods, including the pulsed laser ablation of silicon wafer and the heat decomposition of silanes, or through chemical strategies which usually involve in the reduction of silicon halides by specific reducing agents (e.g. sodium naphthalenide, lithium naphthalenide, lithium–aluminum hydride and zintl salts). In a recent study, Bertucci et al. constructed a microRNA therapeutics-encapsulated pSiNPs with the tumor-homing peptide displaying on the surface (Fig. 5a) to achieve efficient cancer treatment targeting to miR-21, an oncogenic miRNA overexpressed in many tumors [260]. By means of the calcium silicate-trapping chemistry, a significantly high encapsulation efficiency (97 ± 2%) of anti-miR-21 oligonucleotides within pSiNPs (17 wt%) was obtained, and the tumor-targeting pSiNPs displayed a significant growth inhibitory effect on ovarian tumor via down-regulating the expression level of miR-21.

pSiNPs are degradable in organisms, and the produced free silicon atoms can be converted into non-toxic silicic acid, which can be effectively metabolized and eliminated by the human body. However, the freshly prepared pSiNPs are prone to rapid degradation in aqueous medium, and thus post-modifications (e.g. surface functionalization with polymers) are needed to improve their stability. For instance, Kafshgari et al. constructed a chitosan-decorated pSiNP system for TO delivery [261], where the chitosan coating could significantly improve sustained oligonucleotide release and enhance the internalization of oligonucleotide-loaded pSiNPs across the cell membrane. Furthermore, the biocompatibility and non-inflammatory properties of pSiNPs were well maintained through in vivo evaluations. In another study [262], Tong et al. established a polyethyleneimine (PEI)-capped pSiNPs system to achieve high-capacity loading for delivery of siRNA, targeting to the gene of multidrug resistance-associated protein 1 (MRP1), an overexpressed chemoresistance-associated biomarker in glioblastoma multiforme (GBM). Optimized siRNA release (70% released over 48 h) and effective knockdown of MRP1 expression in GBM by 30% were observed. The authors demonstrated that MRP1-siRNA loaded pSiNPs could successfully silence MRP1 in GBM tumors 48 h post-injection and a significant reduction of GBM proliferation was observed, which may be mediated by the decrease of MRP1 transmembrane transport and subsequent leading to cell cycle arrest.

Except directly using as the TOs delivery vehicle, pSiNPs can also be combined with other NP formulations to overcome the shortages of each single formulation. For example, it has been reported that the fusogenic liposomes, which can directly fuse with cell membrane to avoid insufficient endocytotic sequestration and subsequent lysosomal degradation, generally suffer from low loading capability of TOs and non-negligible leakage during circulation in vivo. In order to overcome this obstacle, Kim et al. designed a novel delivery system, termed fusogenic nanoparticles (FNPs) [263], which harnessed together a fusogenic lipid and a pSiNP core, the latter of which could efficiently encapsulate siRNAs via the Ca^2+^ precipitation strategy with minimal leakage [264]. FNPs demonstrated a receptor‐independent endocytosis cell uptake mechanism, and showed great potential for sensitizing cancer cells to chemotherapy in gene therapy and immunotherapy in polarizing macrophages towards the tumorigenic M1 phenotype.

#### 4.4.2 Mesoporous silica nanoparticles (MSNs)

Silica is an oxide form of silicon with the chemical formula SiO_2_. SiO_2_-NPs are usually synthesized by the hydrolysis of the tetraethyl orthosilicate (TEOS) in an alkaline medium. Using the commercially available alkoxysilanes or halosilanes, the surface of SiO_2_-NPs is facile to functionalize with amino, mercapto, epoxy or acryl groups to facilitate the immobilizations of fluorophores, targeting moieties or drugs. By adding surfactants (such as cetyl trimethylammonium bromide), micelle forming type materials, polymers, or other dopants to the synthesis solutions, mesoporous silica nanoparticles (MSNs) can be obtained [265]. It has been demonstrated that the increased delivery efficiency of MSNs can be achieved by tailoring their size, shape and external surface functionalization [266]. A significant advantage of MSNs in TO delivery is their controllable porous structures and surface functionalization, endowing them with extremely high TO-loading capacity. Taking siRNA for example, Möller, et al. once reported a core-shell mesoporous silica nanoparticle system with multifunctional polymer caps, where they demonstrated a loading capacity of siRNA could reach as high as 380 μg mg^−1^ [267].

Recently, Wang et al. developed a novel MSNs platform, termed iMSNs (Fig. 5b) [268], in which the large internal surface area and pore volume make it easy to carry various therapeutics. On the one hand, stabilized structures endowed iMSNs a high loading efficacy for co-delivery of mRNA and siRNA via modification with a surface lipid layer, which conjugated to the iRGD peptide. On the other hand, a photosensitizer, ICG, was loaded into the iMSNs for *in situ* generations of ROS to realize NIR triggered cytoplasmic RNA release. Thereby, this co-delivery nanoplatform improved therapeutic effect by silencing multiple target genes and cellular pathways of tumor cells simultaneously acted on eliminating the primary tumor and inhibiting the metastatic process. In addition to cellular generation of ROS, cytosolic Ca^2+^ is also a vital regulator of signal transduction in various cancer processes containing proliferation, tumor genesis, and migration [269,270]. The controlled increase of cytosolic Ca^2+^ concentration through the plugging of the T-type Ca^2+^ channel with antagonists or siRNA can inhibit cancerous proliferation, while the specific delivery of antagonists or delicate siRNA to tumor site remains an inevitable challenge. Wang et al. fabricated the mesoporous silica nanocapsules (MSNCs) for cocktail delivery of both doxorubicin (DOX) and siRNA targeting T-type Ca^2+^ channels concurrently (Fig. 5c) [271]. They demonstrated that the accumulation of intracellular drug concentration led to the decrease of cytosolic Ca^2+^ concentration, downregulated expression of Ca^2+^ channel and specifically induced of G_0_/G_1_ arrest of the cell cycle, finally exhibited an extraordinary synergistic therapeutic effect for multidrug resistant breast cancer. Therefore, the delivery of TOs acted on T-type Ca^2+^ channels could be a strategy with great potential for multidrug resistant cancer treatment with MSNCs.

In another report, silica nanoparticles demonstrated a well performance in overcoming the shortages of nucleic acid nanoparticles (NANPs) when considering their targeted delivery in vivo. NANPs have been identified as a novel but promising scaffold in incoprorating TOs for anticancer purpose due to their excellent capability of tailoring DNA/RNA sequences in a programmable manner. However, the susceptibility of NANPs to enzymatic degradation as well as their inability to cross biological membranes seriously limited their clinical transition. To overcome these problems of NANPs, Juneja et al. combined NANPs with MSNPs via the electrostatic association, and the formed nanoconstructs could not only effectively deliver NANPs to their targe sites but also synergize with other therapeutic agents to achieve enhanced anticancer effect [272].

#### 4.4.3 Metal-organic framework

Mental-organic framework (MOF), a type of porous hybrid materials establish from metal irons or clusters bridged by organic ligands, has been widely developed and exploited in diverse fields, including gas storage and separation [273,274], catalysis [275], biosensing [276], and biomedicine [277,278]. When exploited as the drug delivery platform, MOFs show promising prospects and advantages for the treatment of cancer, which include but not limited in the following aspects: i) the large surface area and precisely controllable porous structure endow the MOFs with a high drug loading capacity; ii) the structure of MOFs can be well adjusted to achieve stimuli-responsive drug release with a high specificity in tumor tissues; iii) MOFs are biodegradable and have shown good biocompatibility in a numerous studies [279,280]. When used as the drug delivery vehicle of TOs, MOFs show extra advantages, e.g. the metal elements that form the MOF structure may also be an active component synergistically taking part in the therapeutic action of TOs. Therefore, MOFs have received a much broader attention in recent years, as an alternative delivery system to the existing nanocarriers in the targeted delivery of TOs [[281], [282], [283], [284]].

It should be noted that the traditional MOFs used for loading small molecular drugs were no longer effective in encapsulating TOs within their internal pores, due to the macromolecular nature of TOs [285]. To overcome this obstacle, Michelle et al. [286]. recently established a highly porous zirconium-based MOF structure, named NU-100, an optimal structure selected by molecular simulations, offering a porosity compatible with siRNA. The authors demonstrated the successful loading of the siRNAs within the internal pore of MOF, not on the surface of the framework, via the fluorescence-lifetime imaging microscopy. In addition, the combined encapsulation of species that were able to open up endosomes with siRNA@MOF complex was proved as an effective way to deliver siRNA to cytoplasm and achieved sufficient gene knockdown. Different from the method of synthesizing large TO molecules and infiltrating them into the MOFs with large pores, Liu and coworkers developed a facile and versatile strategy to prepare MOF‐based biomimetic co-delivery system that can greatly increase drug loading efficiency [287]. The antisense oligonucleotides that target the antiapoptotic protein Bcl-2 (G3139) and doxorubicin were co-assembled into a uniform NP formulation (DOX/Fe‐G) with the aid of Fe^II^ ions. Subsequent biomineralization of the DOX/Fe-G surface with a thin shell of ZIF-8 MOFs was used to produce the final core-shell form of the nanoplatform (DOX/Fe-G@Z), which exhibited effective both in vitro and in vivo. Subsequently, the thin shell of ZIF-8 MOFs were mineralized on the surface of DOX/Fe‐G to produce the final core-shell nanostructures (DOX/Fe‐G@Z), which exhibited effective tumor growth inhibition both in vitro and in vivo. The authors emphasized that the current straightforward biomimetic approach could greatly engender broad opportunities of MOF for practical uses.

Apart from the physically encapsulation method, DNA molecules can also be loaded in MOFs via covalent conjugation [288] or coordination chemistry-based strategies [289,290]. In 2014, Mirkin group reported the first MOF-DNA conjugate [288], which was realized by the Cu-free strain promoted click reaction between the azide-functionalized zirconium based framework, UiO-66-N_3_ and the dibenzylcyclooctyne-modified DNA. Compared with bear MOF particles of comparable size, DNA conjugation increased MOF stability in aqueous NaCl and enhanced cellular uptake efficiency. Later on, they developed a more general strategy to functionalize MOF nanoparticles with oligonucleotides at high density through modifying the terminal of oligonucleotide with a phosphate [289], which could coordinate with a series of unsaturated metal nodes on their respective MOF structure. Note that the modification of oligonucleotides produced additional cost and may interfere the function of oligonucleotides, particularly when they are active drug molecules. To overcome this limitation, Wang et al. constructed a method based on intrinsic coordination for preparation of MOF-DNA complex from naïve oligonucleotides, which could be adsorbed on MOFs through multivalent binding between the phosphate backbone and zirconium centers (Fig. 5d) [290]. Moreover, biomineralizing MOF-DNA with a calcium phosphate (CaP) exoskeleton could endow the nanoconstruct with physiologically-stimuli-responsive DNA-release properties: at acidic endo/lysosomes, CaP dissolved under acidic pH conditions, generating phosphate ions which could efficiently display DNA from MOFs. Using immunostimulatory CpG as the model payload, MOF-CpG@CaP displayed high cellular uptake and organelle specificity, as well as spatiotemporal control of the immune response triggered by Toll-like receptors (TLR).

DNAzymes, a type of synthetic single-stranded DNA molecules that can catalyze some biological reactions similar to RNAzymes and proteins [291], have recievedgreat attention in the treatment of cancers owing to their capabilities of inhibiting multiple tumorigenic processes via the catalytic cleavage of oncogene targets when supplied with enough specific cofactors [292]. However, the low efficacy of cellular uptake and inadequate cofactor supply prevents the in-depth development of DNAzyme as an effective drug candidate to clinic. To address these shortcomings, Wang et al. [293]. designed a self-sufficient nanosystem for DNAzyme delivery based on the zeolitic imidazolate framework-8 (ZIF-8) nanoparticle, a zeolitic imidazolate framework, capable of disassembling at acidic endo/lysosomes accompanied by the continuous release of the DNAzyme payloads and the cofactor Zn^2+^, and finally promoting the DNAzyme-mediated gene silencing. This study showed a good example of designing smart gene delivery systems based on the specific intracellular biocatalytic reactions that have important therapeutic relevance, representing a new direction in the drug design. In another study [294], the same group designed a DNAzyme-based delivery system, called DNAzyme nanosponges, consisting of a DNAzyme–substrate scaffold fabricated by the rolling circle polymerizations and several ZnO nanoparticles. Synergistically enhanced therapeutic performance was achieved by the pH-responsive dissolution of ZnO into Zn^2+^, which further mediated nonviolent DNAzyme-catalyzed cleavage of DNA scaffolds and thereby realizing precise and effictive drug delivery.

Similar to other types of nanocarriers, endosomal escape should also be carefully considered in the rational design of TO-loaded MOFs. To realize the endo/lysosomal escape and enhance the TOs delivery to the intracellular target, Lin et al. designed a photo-controllable MOF nanoswitch fabricated from ZIF-8 nanoparticles with the photothermal agent indocyanine green (ICG) encapsulated within the MOF pores and the siRNAs immobilized on the surface through electrostatic adsorption [295]. Upon mild laser irradiation, large amount of siRNA was released intracellularly accompanied by the dissociation of MOF complex into protonatable 2-methylimidazalo and osmotic rupturing Zn^2+^ ions, which cooperatively led to endo/lysosomal rupture. The nanoswitch displayed a high degree of spatiotemporal control of gene transfer for cancer site-specific RNA interference. Interestingly, the dissociation of MOF complex caused the recovery of ICG fluorescence which facilitated the real-time monitoring of siRNA delivery.

In summary, the physicochemical characteristics of MOFs as well as their variability of coordination metal ions and organic ligands make them promising nanocarriers for TOs delivery. High drug load efficacy and stimuli-responsive drug release can be achieved through MOF structural optimization and rational design. Meanwhile, TOs delivery based on MOFs has attracted more and more interest in the combination of gene therapy with other therapeutic strategies, e.g. chemotherapy, photodynamic therapy, photothermal therapy, as well as immunotherapy owing to the well-controlled porous structure of MOF.

### 4.5 DNA/RNA nanoassembly

Due to their programmable and predictable capability of hybridization with their complimentary sequences, DNA/RNA molecules have attracted more and more interest in the construction of nanoassembly for the smart delivery of TOs. Strategies developed for fabricating DNA/RNA nanoassembly mainly include DNA origami [296], single-stranded tile (SST) DNA nanostructures [297], and single-stranded (ss) DNA/RNA origami [298]. Importantly, TOs themselves can not only exhibit the therapeutic effect but also serve as the part of building block in the fabrication of DNA/RNA nanoassembly, increasing their targeted efficacy. In brief, DNA/RNA nanoassembly as an ideal TOs delivery nannocarriers has the following characteristics. Firstly, the excellent biocompatibility of the DNA/RNA entities; Secondly, the vigorous self-assembly capability through Watson and Crick base-pairing interactions; Thirdly, designable and controllable precise structures with addressable modification sites; Fourthly, the facile functionalization methods for targeting ligands and TOs. Up to now, numerous DNA/RNA nanostructures have been applied in the delivery of TOs like siRNAs, mRNA, and immune modulators for gene therapy, target therapy, and immunotherapy.

#### 4.5.1 siRNA-hybridized DNA nanoassembly

**Fig. 6:**
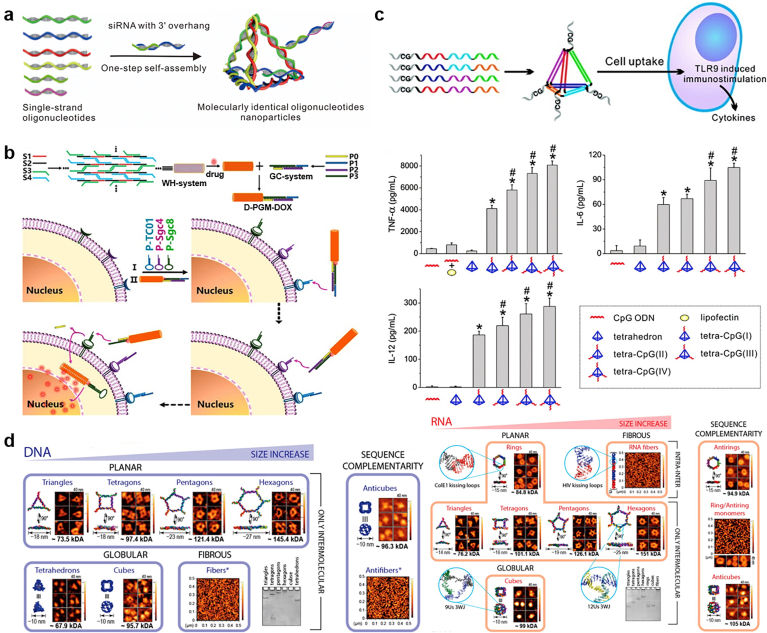
**TOs delivery based on the DNA/RNA nanoassembly. (a)** Self-assembled DNA tetrahedral nanostructure for targeted siRNA delivery (arrow head represents 5 end of the nucleic acid strand). Reproduced according to Ref. [301].; **(b)** Stepwise self-assembly of D-PGM for three-receptor-mediated cell identification and targeted cancer therapy. Reproduced with permission [305], Copyright 2020, American Chemical Society; **(c)** Self-assembly of CpG-bearing DNA tetrahedrons and their multivalent immunostimulatory effect evaluations. Reproduced with permission [306], Copyright 2011, American Chemical Society. **(d)** Nucleic acid nanoparticles (NANPs) differing in size, shape, composition, connectivity, and sequence complementarity for immunological recognition investigations. Reproduced with permission [312], Copyright 2018, American Chemical Society.

siRNAs as a class of potent therapeutics for various cancer treatment from aberrant gene expression, remain to be precluded from cell permeability and resistance to degradations [299]. Despite an appropriate manner to overcome the inevitable leakage of synthetic siRNA and off-target toxicity, the current intracellular generation (i.e. *in situ* production in target cells) of siRNA by a spontaneous collision between complementary strands confined the assembly efficiencies owing to the lack of controllability and the slow reaction rate [300]. Lee et al. designed a DNA tetrahedron nanoformulations for site-specific hybridization of siRNA [301]. Due to the addressability of the DNA tetrahedron nanostructures which have a nick site in the middle of each edge thus could be further used to tether siRNA strands (Fig. 6a). Using such a self-assembled DNA nanostructure, the sizes, the spatial orientations as well as densities of targeting ligands on the surface of NPs could be precisely controlled, providing an efficient tool for screening the best formulation for optimal cancer therapy. Ren et al. designed a cationic folic acid modified polyethyleneimine capped DNA nanoplatform that constructed by two pairs of DNA/RNA hybrids alternately hybridized to adjacent sites on a DNA scaﬀold [302]. miRNA in the cytoplasm of cancer cells initiated the generation process of siRNA which called DNA templated synthetic reaction, further lead to the hybridization of two anchored DNA strands and continuous siRNA generation without the involvement of exotic enzymes and catalyst, while the precise arrangement sequences guaranteed the high reaction efficiency. This mRNA triggered DNA nanoplatform showed highly efficient siRNA *in situ* generation and enhanced inhibition of miRNA-specific tumor cells, thus can be easily generalized for precise gene therapy.

#### 4.5.2 Aptamer-hybridized DNA nanoassembly

Due to the high-specificity of aptamers in recognizing targeted cells, incorporation of aptamers to the DNA nanoassembly has become a common strategy to increase the targeting efficiency of these nanocarriers. In one earlier study, Wu et al. constructed a poto-crosslinked DNA nanogel (AptNAs), the framework of which consisted of sgc8-aptamers targeting leukemia cells, ASOs targeting multidrug resistance genes, and DNA building blocks [303]. Due to their programmable design in molecular level and excellent selectivity in targeted recognition and transportation, AptNAs displayed a highly-specific toxicity against cancer cells and a significantly down-regulated expression of drug efflux pump protein P-gp, which synergistically enhanced the anti-cancer effect. Later on, the same group reported another DNA nanogel for targeted gene regulation. Different with previous one, the DNA nanogel was crosslinked via a disulfide linking unit with two different Y-shaped building units with sticky sequence distributed at the terminal of each arm [304]. Aptamers targeting A549 lung cancer cells, ASOs targeting c-raf-1 mRNA (a cancer-associated biomarker) and DNAzymes targeting lung-cancer-proliferation-associated matrix metalloproteinase-9 could be hybridized to the DNA framework in a site-specific manner. The formed DNA nanogels could sense the intracellular GSH, resulting in the release of ASOs and DNAzymes to exert their roles in cancer cell inhibition.

Recently, a combination of different signaling circuits consisting of diverse aptamers has also shown great potential in tumor cell-speciﬁc therapeutic applications. Nevertheless, due to the ingenious molecular structure of combinational logic circuits and the highly complex 3D nanostructure, such combinatorial molecular circuits based on multiaptamer have not been applied to DNA nanostructures for drug delivery of cancer treatment, especially for high drug loading nanocarriers with tumor-specific targeting. Ouyang and coworkers developed a triple-receptor targeting precision-guided missile DNA nanovehicle (D-PGM) consisting of specific recognition (GC) and drug carrier (WH) compartment (Fig. 6b) [305]. The GC is a DNA logic nanocircuit composed of three self-assembled ssDNA partially complementary to various aptamers in a highly organized manner, while the WH is a rod-shaped 3D DNA origami assembled from four oligonucleotides as drug carrier. More accurate and effective drug delivery achieved under similar cells conditions when the GC binds to three aptamers and dissociates in an organized manner compared to traditional single or dual targeting nanovehicles. Thus, such a precise triple-receptors based DNA nanostructure provided enhanced diagnostic and therapeutic accuracy and represented a potential strategy for personalized treatment of cancer.

#### 4.5.3 Immunostimulatory oligonucleotides-incorporated DNA nanoassembly

Fan's group designed a DNA tetrahedron structure as a nanocarrier for delivery of immunoregulatory oligonucleotides. Due to the advantage of the programmability of DNA tetrahedron structure, they can selectively connect different numbers of CpG motifs to one DNA tetrahedron structure (Fig. 6c), which significantly enhanced the immunostimulatory effect of CpG because of the multivalent interactions between CpG and the toll-like receptor 9 (TLR9) [306]. Recently, Christopher et al. proposed a replicable ssRNA origami (RNA-OG) technology, through which a long RNA molecule can be programmed to self-assemble into various custom-shaped nanostructures. For instance, polyinosinic/polycytidylic acid (PolyIC), a well-known dsRNA analog, have shown efficacy in cancer immunotherapy through Toll-like receptors 3 (TLR3) triggered innate immune and entered clinical trials [307]. Nevertheless, these analogs-induced high concentrations of type-I interferons (IFNs) lead to systemic toxicity cannot be ignored. Recently, Qi et al. reported RNA origami nanostructures produced from a single strand RNA with high accuracy and yield while do not require as many short synthetic DNA strands as traditional DNA origamis [308]. They demonstrated that achieving a continued antitumor immunity that depends on the presence and levels of CD8^+^ T and NK cells. Compared with PolyIC administration, the levels of IFNs produced systemically is much lower as well as the systemic toxicity, and the nature of immunosuppression in the peritoneal cavity can be reduced. The RNA origami could be developed as a powerful and safe nanomedicine for cancer immunotherapy.

Linear short hairpin RNAs (shRNAs) have exhibited enhanced stability and induced immune response in vivo compared to siRNA, which is generally used for multidrug delivery [309]. The multiple mechanisms of drug resistance like drug efflux and inhibition of apoptosis, severely limit the effective intracellular release of nanodrugs in cancer cells, resulting in insufficient therapeutic effect. At the same time, owing to the excellent abilities of multidrug co-loading and editable size and morphology of DNA nanostructures, Liu and coworkers fabricated a precisely self-assembled DNA origami containing chemotherapy drug loading nanostructure and two kinds of linear shRNAs outside [310]. The DNA nanoplatform realized the co-delivery of various drugs and eventually achieved a synergistic anticancer effect among diverse cargoes: shRNA silenced the multidrug-resistant associated genes which reduced the drug eﬄux thus led to the accumulation of chemodrugs and induced apoptosis of tumor cells, while chemodrugs played intrinsic cytotoxicity. By integrating TOs with DNA nanoplatform and collaborating with other drugs, it is possible to make the treatment effective.

Despite tremendous advances in the development of DNA/RNA nanoassembly, its immunotoxicity, one of the major obstacles to traditional TOs clinical transformation, has not been fully characterized. Guo and coworkers demonstrated that the immunogenicity of RNA nanoassembly is depending on the size, morphology, and specific sequence. Importantly, they found RNA polygons that do not extend at the vertices are immune inert. Thus, the immunogenicity of RNA nanoassembly can be adjusted to produce minimum immune response as a safe therapeutic nanovehicle, or a strong immune response as a vaccine adjuvant for cancer immunotherapy [311]. These conclusions were further confirmed by Hong and his coworkers [312]. In their study, they systematically studied the immune recognition capabilities of representative DNA/RNA NANPs with different parameters (Fig. 6d), and found that type III interferons (IFN-III) is additional biomarkers of nucleic acid nanoparticles (NANPs) after their internalization by phagocytic cells, compared to traditional DNA/RNA nanoassembly. Furthermore, the immunomodulatory effect of NANPs relies on multiple physicochemical properties, including size, structure (planar or three-dimensional construction), composition, and connectivity. Predictably, NANPs can be programmed as a prototype of specific molecular language for communication with immune system and regulation of immune response.

### 4.6 Extracellular vesicles

**Scheme 2:**
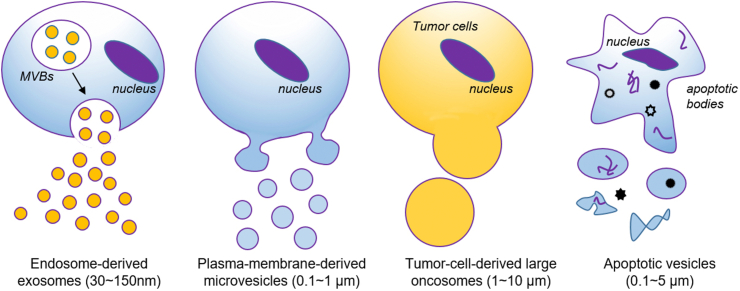
Schematic illustration of subtypes of extracellular vesicles.

Extracellular vesicles (EVs) are cell-derived membranous particles that can be naturally released from a cell, but unlike a cell, EVs cannot replicate. EVs are present in various body fluids [313], and are crucial carriers that participate in intercellular communication during tissue homeostasis and repair under physiological conditions [314,315]. According to the size and origin, EVs are classified in to four main subtypes (Scheme 2), including endosome-derived exosomes, plasma-membrane-derived microvesicles, large oncosomes (tumor-derived large vesicles), and apoptotic bodies [316,317]. The contents of the cargos loaded in EVs can distinguish from those in the originating cells, which unveil a selective loading process [318]. Prolonged EVs in circulation can, therefore, act as robust, minimally invasive, specific biomarkers for diagnosing a variety of diseases, including cancers [319,320]. Owing to their low immunogenicity and capacity to cross biological barriers [321], EVs has been identified as a promising vehicle in transporting a variety of cargos, such as lipids, proteins, DNA/RNA species, for cancer treatments [[322], [323], [324], [325]]. Since endogenous RNA species have been reported to be definitively present in EVs, EVs showed superior biocompatibility and delivery efficiency for TOs compared to synthetic nanoparticles [326,327]. Generally, EVs that are utilized for the delivery of TOs in the application of cancer therapy can be categorized in to natural sources and engineered sources.

#### 4.6.1 Natural EVs

EVs can be secreted by almost all cell subtypes, and there are some natural secreted EVs that contain therapeutic RNA/DNA can be used for cancer therapy directly. For example, the mesenchymal stem cells (MSCs) derived miR-143, miR-133b, miR-9-3p rich exosomes can be used for the treatment of prostate cancer, glioma and bladder cancer via their targeting proteins [[328], [329], [330]]. And some nature killer (NK) cell derived EVs that contain miRNAs, such as miR-186 and miR-3607–3p can also be used for neuroblastoma and pancreatic cancer treatment [331,332]. Moreover, the natural derived EVs are also considered as ideal nanocarriers for synthesized TOs owing to their nucleic acid loading capacity and membrane permeability. Based on this, several micRNA mimics and siRNAs have been reported to be loaded into HEK293 and dendritic cells derived exosomes for cancer therapy [320,333,334]. And the most commonly used methods for TOs loading are transfection reagent, ultrasound, freeze-thaw cycle and electroporation [[335], [336], [337], [338]].

#### 4.6.2 Engineered EVs

Compared to natural EVs, the delivery of TOs with engineered EVs are more potential because of their enhanced targeting and customized diversity functions [339]. Recipient cells can internalize EVs through a variety of pathways, including membrane fusion, receptor-mediated endocytosis, lipid-raft-mediated endocytosis or micropinocytosis. It has been reported that the natural targeting ability of EVs to recipient cells is determined by the specific biomarkers (e.g. tetraspanin, integrin) derived from the producer cells [340]. For example, EVs displaying Tspan8 (tetraspanin molecular family)/integrin α4 complex was able to selectively target EVs to cells in the pancreas [341]. Furthermore, unlike negative EVs that interact only with the dendritic cells, CD63-positive EVs mainly target to neuronal and glial cells [342]. While for EVs derived from the human embryonic kidney cells (HEK293T), the isolated EVs are absence of integrin markers [343,344], and thus have limited targeting effect towards the recipient cells [345]. To increase the specificity of HEK293T-EVs, Pi et al. decorated EVs with an arrow-shaped three-way junction RNA nanoparticles (pRNA-3WJ), containing a cholesterol for membrane anchoring, a fluorophore for imaging, and a ligand for tumor cell targeting [346]. The resulting ligand-displaying EVs were able to specifically deliver siRNA to cells, and showed effective inhibition of tumor growth in the three cancer models studied in this research.

As for drug delivery vehicles, the potential interaction between the natural bioactive payloads and therapeutics may interfere with the desired therapeutic effects. To address these hurdles, several strategies, such as genetic manipulation and functionalization after secretion, have been developed recently [[347], [348], [349], [350]]. Nevertheless, these additional modifications, in some ways, compromise the very crucial components in exosome-cell interactions and drug delivery [351]. On this account, Yerneni et al. proposed a scalable, practical approach for rapidly and efficiently manipulating engineer exosomes with oligonucleotide conjugates [352]. With a conjugated cholesterol moiety, the single-stranded DNA (ssDNA) can be tethered to an external lipid bilayer of exosomes, while the complementary DNA can easily attach to various functions such as reactive functional groups and small molecules. The confocal micrographs showed that the aptamer AS1411-tethered exosomes preferred to bind to nucleolin on MIAPaCa2 (human pancreatic cancer cells) cell surface rather than HEK293 (normal cell), and was thereby able to circumvent the inhibitory effects of heparin and methyl-β-cyclodextrin resulting in altering the specific targeting capability. Meanwhile, ssDNA tethered exosomes achieved spatially restricted apoptosis in PCI-13 cells in virtue of bioprinting through live/dead staining. The oligonucleotide tethers have shown their facile, robust, and general versatility in the augmentation of the extracellular vesicle that is independent of its cell origin, and was conducive to the clinic transformation of gene-drug delivery based on exosome.

### 4.7 Imaging-guided drug delivery systems (IGDD)

Imaging-guided drug delivery (IGDD) systems, referring to the combination of drug targeting and imaging, have emerged as the essential component of personalized cancer treatment. IGDD can be employed to visualize and quantify the biodistributions of drugs, and to non-invasively assess their therapeutic efficacy. In the field of TOs delivery, IGDD systems can significantly improve the targeting delivery efficiency of TOs and make the cancer treatment more precise. In the following section, we will introduce three typical IGDD systems based on the nanoparticles or nanoassembly that can not only behave as the drug vehicle, but also display imaging properties.

#### 4.7.1 Iron oxide nanoparticles

Iron oxide nanoparticles (IONPs) are a class of inorganic nanoparticles with interesting properties like small volume, large surface area, magnetic and superparamagnetic [353]. IONPs can be formed by placed in a specific magnetic field, which allows heat to be absorbed by electromagnetic current waves in an alternating magnetic field. The unique properties of IONPs have been exploited for cancer diagnostics, as well as drug delivery [354]. As for delivery of TOs, the targeting and efficacy remains a significant obstacle to explore their clinical contex. Jiang et al. developed a magnetic siRNA delivery nanosystem composed of a lipid-like shell (termed lipidoid) and an ION core [355]. They found that the size of lipidoid-coated IONs affected the delivery efficacy of DNA, while there is no obvious difference in the delivery of siRNA. In addition, enhanced targeting delivery of TOs was achieved with the external magnetic field compared to commercial Lipofectamine 2000. The lipidoid IONs delivery platform proposed here provides an optimal strategy for magnetic guided targeted delivery, and the opportunity to combine the advantages of gene therapy with MRI and magnetic hyperthermia.

**Fig. 7:**
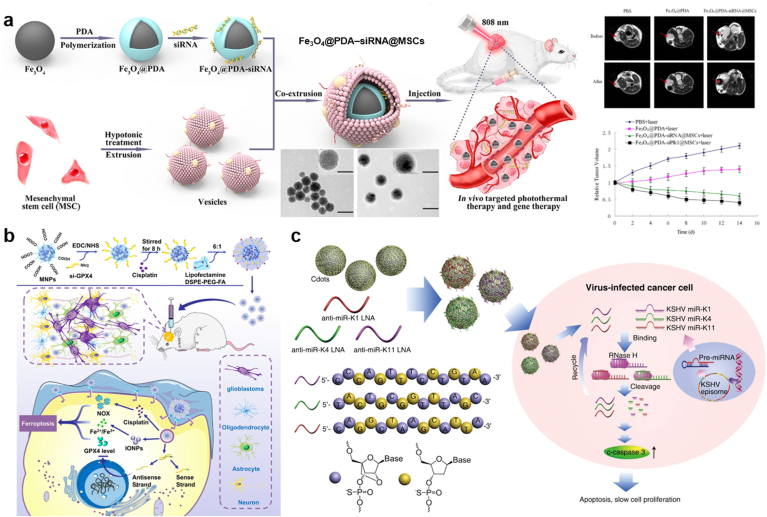
**TOs delivery based on imaging-guided drug delivery systems. (a)** MSC membrane-coated Fe_3_O_4_@PDA–siRNA NPs for the tumor-targeting siRNA delivery. Reproduced with permission [362], Copyright 2018, American Chemical Society; **(b)** si-GPX4/Pt-codelivery IONPs platform for the synergistic treatment of glioblastoma. Reproduced with permission [366], Copyright 2020, American Chemical Society. **(c)** Targeting Kaposi's sarcoma-associated herpesvirus (KSHV)-encoded miRNAs by CDs-mediated delivery of locked nucleic acid (LNA) for treating KSHV-induced cancers. Reproduced with permission [375], Copyright 2020, American Chemical Society.

In recent years, membrane-derived vesicles have been used as natural analogs of liposomes for drug delivery. The capsule has a component of the source cell membrane, which makes it inherently biocompatible and slightly immunogenic in vivo. Several studies have proposed some biomimetic delivery platforms from various cell membranes, including stem cells [356], platelets [357,358], leukocytes [359], and cancer cells [360,361], to apply in personalized biomedicine for cancer treatment. Mu and coworkers employed the first mesenchymal stem cells membrane-coated IONPs for siRNA delivery. IONPs@PDA exhibited favorable photothermal capability and high siRNA loading efficiency, which maintained the magnetic resonance imaging (MRI) inherited and photothermal effects after membrane coated (Fig. 7a) [362]. The membrane-coated nanovehicles were endowed with excellent blood circulation and remarkably tumor targeting property. siRNA induced Plk1 gene knockdown significantly inhibited the bioactivity of endogenous Plk1 gene and cause obvious ablation of tumor, therefore achieved MRI-guided gene therapy and photothermal therapy.

Compare to the non-invasive trigger, the endogenous stimulatory, hypoxia, plays a key role in solid tumor development and resistance to therapy, providing compelling target sites for arresting tumor progression [363]. For instance, the monocarboxylate transporters (MCTs) upregulate in primary and metastatic tumors owing to the excessive intracellular concentration of lactic acid from active glycolysis. Besides, the elevated reactive oxygen species (ROS) in tumor cells make it more vulnerable to oxidative assault. These hallmarks present a novel direction for combinatorial cancer therapy. Liu and coworkers constructed amorphous iron oxide (AIO) nanoparticle-based siRNA delivery nanovehicles (siRNA@NPs) for co-targeting two cancer hallmarks [364]. siRNA loaded AIO NPs via electrostatic interactions are coated by biocompatible lipid and responsive to acidic pH after cellular uptake, further, the released iron ions induced endosomal membrane oxidation through the osmotic pressure in which leads to the effective endosomal escape of siRNA. Hence, the intracellular lactate/H^+^ accumulated via siRNA-mediated MCT4 silencing while facilitated more H_2_O_2_ production triggered by hypoxia, to amplify the oxidative damage procession: reacted with iron ions, H_2_O_2_ produced highly reactive and toxic •OH which completely aggravated oxidative stress of tumor cells and subsequent cancerous cell death. This innovative nanosystem achieved effective gene silencing and also exhibited MRI-mediated tracking of siRNA, which prompts the applications of TOs in theranostic nanomedicine.

In addition to the capability of directly transporting drug payloads to the desired targets, IONPs can also act as an effective and easily available carrier for the intracellular delivery of iron (Fe^2+^/Fe^3+^), which can take part in the iron-dependent programmed cell death through a process called Fenton reaction, an innovative strategy that has been identified recently in the treatment of malignant cancers [365]. Zhang et al. designed a targeting system based on the porous IONP that could deliver irons, chemotherapeutic cisplatin (Pt), and the siRNA targeting glutathione peroxidase 4 (si-GPX4) for the elimination of glioblastoma multiforme (GBM) [366]. The co-delivery system displayed a highly efficient synergistic effect (Fig. 7b): on the one hand, Pt induced apoptosis and simultaneously produced H_2_O_2_, together with the irons (Fe^2+^/Fe^3+^) released from IONP significantly increased the ROS generation (remarkably •OH) via the Fenton reaction within GBM cells; while on the other hand, si-GPX4 can effectively know down the negative regulator (GPX4) acted on the ferroptotic process. Finally, the co-delivery system showed an outstanding therapeutic effect against GBM model in vivo.

#### 4.7.2 Carbon dots (CDs)

Carbon dots (CDs), also called carbon quantum dots or carbon nanodots, are organic compounds-derived carbon-based materials with the size below 10 nm. As emerging luminescent materials, CDs have shown great potential in the construction of multimodal imaging agents as well as in the delivery of oligonucleotides because of their remarkable photo-stability, high quantum yield, tunable emission, broad excitation spectra, cost-effective synthetic method, and low toxicity [[367], [368], [369]]. When used as the delivery vehicle for TOs, the surface of CDs must be modified with specific moieties for TOs’ binding. By modifying the CDs surface with cationic polymer, like polyethylenimine (PEI), poly-l-lysine, and poly(amidoamine) (PAMAM) [370], CDs exhibit water solubility and accomplish surface passivation, as well as stable binding efficacy of TOs which further released after internalization by the targeted cells [371,372]. For instance, Kim et al. synthesized fluorescent PEI-passivated CDs (CDs-PEI) for delivery of siRNA targeting the breast cancer cells [373]. The CDs-based RNAi displayed a high efficacy in gene knockdown both in vitro and in vivo and a clear intracellular tracking of the gene delivery process could be approached without additional fluorescent conjugates.

It has been reported that 15% of human cancer are associated with the virus-infections [374]. Targeting oncogenic viruses-encoded miRNAs represent a very promising therapeutic approach. Recently, Ju et al. used CDs as the carrier to load the locked nucleic acids (LNA)-based oligonucleotides, through electrostatic interaction, for specific knockdown of virus miRNAs (Fig. 7c) [375]. LNA oligonucleotides with an increased binding affinity exhibited the ability to re-induce the degradation of target miRNAs, leading to enhanced apoptosis and slowing down the process of proliferation. Moreover, since cancer viruses reside in viral cancer cells rather than in normal cells, targeting miRNAs of oncogenic viruses is an ideal approach for combating virus-induced cancers, and provided the specific delivery of TOs without additional modification of targeting ligands.

Apart from luminescence-based imaging, CDs-based gene carriers can also be designed to achieve multi-modal imaging. For example, He et al. fabricated novel Gd-doped CDs grafted with cationic polymers to achieve the combined gene delivery and magnetic resonance (MR)/fluorescence dual modal imaging [376]. The multifunctional CDs showed much higher transfection efficiency than PEI 25 kDa and could realize spatiotemporal monitoring of the gene delivery process via fluorescence imaging. In addition, the Gd-doped CDs displayed an improved MR imaging performance, where the longitudinal relaxation rates were at least 3- times higher than that of the clinically used Gd reagent (Gd-DTPA).

#### 4.7.3 Aggregation-induced emission (AIE) nanodots

Discovered by Tang's group, aggregation-induced emission luminogens (AIEgens) have shown great potential in the real-time monitoring of biological dynamics [377]. AIEgens are non-fluorescence when they are dispersed in the solution in a single molecular state, while they will display strong fluorescent emission upon forming aggregates because of the restriction of intramolecular rotation. Nowadays, various AIEgens-based nanoscale fluorescent probes, namely AIE nanodots, have been widely developed as the novel IGDD system due to their high sensitivity and spatio-temporal resolution [378,379]. As for the TOs delivery, AIE nanodots can either be fabricated by incorporating AIE dyes to the preformed nanoparticles or through self-assembling AIE-moiety-containing amphiphilic conjugates [[380], [381], [382], [383]].

In one earlier study, Hu et al. reported the first AIE nanodots system for siRNA delivery [381]. The AIE nanodots were prepared by physically encapsulating AIE dyes to mixed polymeric micelles from Pluronics F127 and mPEG-DSPE, which were further decorated with a positively-charged polymer PAH for siRNA loading. The authors monitored the intracellular delivery of siRNA by means of AIE-nanodots and demonstrated that the AIE-nanodots@PHA/siRNA complex could significantly suppressed the expression of the pancreatic cancer-associated K-ras gene. To overcome the endo/lysosomal entrapment of gene vectors, Yuan et al. designed a novel AIE nanodot assembled from a ROS-responsive co-polymer [380], which consists of a photosensitizer TPECM with AIE characteristics and DNA-binding oligoethylenimine co-conjugated in the polymer backbone via the photocleavable aminoacrylate linker. When incubated with cells, the nanodots/DNA complex were mostly found in the endo/lysosomes. While upon visible-light irradiation, TPECM produced ROS, leading to the disruption of the endo/lysosomal membranes and cleavage of the polymer which thus caused the DNA release for transcription. In a recent study, the same group reported another type of AIE-nanodots which also demonstrated a ROS-responsive endo/lysosomal escape properties [382]. Different with previous study, here they covalently immobilized the anti-apoptotic Bcl-2 antisense oligonucleotides (OSA) to the AIE nanodots via the Click reaction. AIE-based SNAs enabled the real-time monitoring of the biodistributions of OSA in vivo. Upon light irradiation, the intracellular SNAs dissociated and the released OSA caused the degradation of the Bcl-2 mRNA. In vivo evaluations indicated that combined treatment of AIE-based SNAs with light significantly inhibited the tumor growth with no effect to the healthy tissues. Recently, Cheng et al. synthesized an amphiphilic peptide-AIEgens conjugate for simultaneously visualizing and delivering antisense oligonucleotides (ASO) to nucleus [383]. The peptide sequence contains three parts: a peptide segment targeting integrin-overexpressed cancer cells, a nuclear localization signaling peptide for nuclear-targeting delivery of ASO, and a positively-charged membrane-penetrating peptide for both ASO-loading and endosomal escape purposes. The multifunctional peptide-AIEgens assembly not only enabled the visualization of the ASO delivery process in real-time, but also exhibited superior Bcl-2 suppression in targeted cell lines and obvious anti-tumor efficacy in vivo.

### 4.8 Tumor microenvironment responsive TOs delivery

The microenvironment differences between tumor and normal tissues provide a new perspective for controllable release of oligonucleotides within tumor sites. In accordance with several characters of tumor microenvironment (TME), such as the weak acidic tumor matrix, reductive environments, elevated intracellular reactive oxygen species (ROS) and up-regulated expression of enzymes, a variety of smart drug delivery systems that can sense these TME characters have been developed in the past decades [[384], [385], [386]]. In this section, we will give a brief introduction on the recent advances of TME-responsive nanocarriers for TOs delivery.

To modulate the immunosuppressive TME and boost the immune response, Jia et al. prepared an acid-/reduction-dual responsive polyplexes (DRPs), which were composed of the block copolymer mPEG-PLA-PHis-ss-PEI, siRNA against the programmed death ligand-1 (siPD-L1) and the metabolic regulator resveratrol [387]. After being transported to the acidic lysosomes, polyhistine (PHis) was protonated and the structure of DRPs was disrupted. The exposure of the disulfide bonds to the reductive environment led to the simultaneous release of siPD-L1 and resveratrol. Results showed that the DRPs treatments not only upregulated mitochondrial oxidative phosphorylation but also increased the infiltration of CD4^+^/CD8^+^ T cells to tumor tissues by re-shaping the immunosuppressive TME. In combination with siPD-L1, significant anti-tumor effect was observed.

As for the polycations-based TOs delivery systems, when the complex entered cells, effective separation of TOs from the complex is beneficial for hybridization to their target sequences. To achieve this purpose, Liu et al. designed a ROS-responsive charge-reversal DNA delivery system. They synthesized a boronic acid-pendanted quaternary ammonium-containing polymer, namely B-PDEAEA, to complex with DNAs [388]. Upon exposure to ROS, the oxidization of boronic acid groups occurred, followed by a cascade reaction, resulting in the DNA release due to the charge-reversal of polymers from positive to negative. The transfection efficiency of ROS responsive B-PDEAEA polymer is more than 15,000 times higher than that of non-responsive PDEAEA counterparts. In another study, Xin et al. synthesized PEG modified copolymers which also contains quaternary ammonium and boronic acid, named PEG-B-PAEBEA, for co-delivery of miR-34a, a well-known noncoding RNA oncogene targets, and volasertib, an inhibitor of polo-like kinase 1 that is overexpressed in many cancer types [140]. Based on similar ROS-liable dissociation of polyplexes accompanied by the TOs release, PEG-B-PAEBEA significantly upregulated the expression of miR-34a and dysregulated related oncogenes, resulting in efficient tumor growth inhibition.

Matrix metalloproteinases (MMPs), usually high-expressed in the extracellular matrix (ECM) of tumor tissues [389,390], are also excellent targets for the design of TME-responsive drug delivery systems. MMP-2, a protease involved in the degradation of tumor ECM, is relatively overexpressed in almost all tumors. In one earlier study, Hatakeyama et al. developed a PEG-peptide-lipid conjugate for TOs delivery. They used the MMP-2 sensitive peptide GGGVPLSLYSGGGG as the linker between PEG and lipid DOPE to fabricate the envelope-type liposomes (PPD-MEND) [391]. PPD-MEND exhibited a long blood circulation property, while once accumulated in tumor tissues, the peptides were cleaved by MMP-2, resulting in the deshielding of PEG which facilitated the internalization of TOs by tumor cells. The transfection activity of TOs by PPD-MEND was approximately 3 times higher than that of PEGylated MEND without the peptide linker. In another study, Huang et al. fabricated a pH-/MMP-2-dual responsive lipid nanoparticles, the surface of which was modified with the positively-charged cell-penetrating peptide pre-masked by a negatively-charged peptide at neutral pH. These two peptides were linked by the third MMP-2-cleavable peptides. At acidic tumor site, the masking peptides became charge-neutral and separated from the nanoparticle surface by the cleavage of MMP-2. Subsequently, efficient cellular uptake, endosomal escape and gene transfection were successfully achieved [392]. Except MMP-2, other ECM enzymes, such as MMP-9 and hyaluronidase, responsive systems have also been designed to address the challenges of TOs delivery [393,394]. These studies suggest that enzyme-sensitive short peptides are delicate designs in fabrication of TOs delivery for cancer gene therapy.

## 5 Nanocarrier based anticancer oligonucleotide in clinic

Both the supplementary of deficient anti-tumor gene and the silencing of tumor driver gene are pursued as approaches for cancer therapy. While the basic requirement for anticancer oligonucleotide drugs is that the oligonucleotides must be delivered to the target position safely and efficiently. The ideal TOs nanocarriers for clinical use are expected to have the ability of immune evasion, target cell or tissue delivering and high efficiency of cell entry [395]. Despite years of development, the high cytotoxicity and immunogenicity as well as problems such as high cost, limited size and poor loading capability are still the main limitations for viral vectors in the application of cancer therapy. Among the non-viral nanocarries, such as lipids and lipid derived materials, polymers, porous nanomaterials, MOF and many other biomaterials that have been developed for TOs delivery in the past several years, the lipid-based nanoparticles (LNPs) were the most widely studied and have the greatest potential for clinical application [51,63]. In 2018, the first lipid based nucleic acid drug Patisiran has been approved by the US FDA [48]. In addition, there are several other lipids based nanocarriers have been tested in preclinical studies (Table 1, Table 2) [[396], [397], [398], [399]].

**Table 1:** Cancer therapy-associated TOs in clinical trials.

TOs types	Name	Targets	Target disease	Clinical Phase
ASO	AZD9150 [78]	STAT3	Lymphoma	Phase Ⅱ
siRNA	ALN-VSP02 [79]	Kinesin spindle protein and VEGF	Cancer	Phase Ⅰ
	TKM-080301 [80]	Polo-like kinase 1	Hepatocellular Carcinoma	Phase Ⅰ/Ⅱ
	siG12D-LODER [81]	KRAS	Pancreatic cancer	Phase Ⅱ
	DOPC-encapsulated siRNA [82]	EphA2	Cancer	Phase Ⅰ
	Atu027 [83]	protein kinae N3	Pancreatic cancer	Phase Ⅰb/Ⅱa
	mRNA-4157 [84]	Cancer cells	Cancer	Phase Ⅰ

Abbreviations: TOs, therapeutic oligonucleotides; ASO, antisense oligonucleotides; siRNA, small interference RNA; DOPC, 1,2-Dioleoyl-sn-glycero-3-phosphocholine; STAT3, signal transducer and activator of transcription 3; VEGF, vascular endothelial growth factor; KRAS, Kirsten rat sarcoma viral oncogene homolog; EphA2, ephrin type-A receptor 2.

**Table 2:** Nanocarrier-based TO-formulations for cancer therapy in clinical trails.

Delivery system	Name	TOs subtypes	Target	Disease	Clinical phase
LNP	siRNA-EphA2-DOPC [404]	siRNA	EphA2	Advanced solid tumors	Ⅰ
	TKM-080301 [80]	siRNA	PLK1	Advanced hepatocellular carcinoma	Ⅰ/Ⅱ
	DCR-MYC [405]	siRNA	MYC	Hepatocellular carcinoma	Ib/2
	ALN-VSP02 [19]	siRNA	VEGF	Solid tumors	Ⅰ
	lipo-MERIT [406]	mRNA	TAA	Melanoma	Ⅰ
	TNBC-MERIT [407]	mRNA	TAA	Triple negative breast cancer	Ⅰ
	mRNA-2416 [400]	mRNA	OX40L	Advanced solid tumor	Ⅰ/Ⅱ
	Pbi-shRNA-STMN1 [408]	shRNA	STMN1	Advanced liver cancer, solid tumor	Ⅰ
	MTL-CEBPA [409]	saRNA	CEBPA	Liver cancer	Ⅰ
EV	iExosomes [410]	siRNA	KRASG12D mutation	Pancreatic cancer	Ⅰ
AuNP	NU-0129 [411]	RNA	BCL2L12	Glioblastoma multiforme	Ⅰ

**Abbreviations:** LNP, lipid-based nanoparticles; EV, extracellular vesicle; AuNP, gold nanoparticles; EphA2, Ephrin type-A receptor 2; PLK1, serine/threonine-protein kinase PLK1; MYC, MYC Proto-Oncogene; VEGF, vascular endothelial growth factor; TAA, Tumor-associated antigen; OX40L, tumor necrosis factor ligand superfamily member 4; STMN1, human stathmin 1; CEBPA, CCAAT/enhancer binding protein alpha; KRASG12D, GTPase KRas G12D; BCL2L12, Bcl-2-like protein 12.

However, the preferred accumulation in liver of lipid based materials caused by the interaction with apolipoprotein E is the main defect that limits its further application [400,401]. Polymeric materials, such as polyamines, polypeptides, and block polymers are not as clinically advanced for nucleic acid delivery as lipid based materials because some additional challenges related to polydispersity, toxicity and biodegradation [51,402]. Other materials, including cell derived vehicles, gold nanoparticles and DNA/RNA nanotechnology were also used for TOs delivery, but the applications in TOs-based therapies still need to be further explored [401].

## 6 Conclusions and Future Perspectives

In the past decades, as researchers gradually realized that the nanoscale non-viral carriers can be exploited as fascinating delivery systems for oligonucleotides-based therapeutics such as short ssDNA/RNA, as well as siRNA, numerous research projects have been carried out to find the best material and formula to achieve higher delivery and therapeutic effects against cancers. Strategies to enhance TOs delivery and therapeutic effects from molecular modification (chemical, terminal, and ribose sugar modification, etc.) to conjugates (lipid, GalNAc, antibody and aptamer conjugates, etc.) have significantly promoted the clinical development of TOs. However, there is still a long way to the clinical transformation of synthetic TOs. For each type of nanocarrier, there are both advantages and disadvantages (Table 3) in targeted TOs delivery, which should be carefully considered before choosing optimal formulation to achieve the best efficacy. Up to now, only ten TOs are in regulatory approval list of FDA and most of the them are targeting to the liver, one of the central metabolic organs [403]. Versatile nanoscale carriers, as summarized in this review, can improve the stability of TOs, enhance their cell uptake efficiency, increase the cytosolic delivery and achieve sustained drug release via smart design of nanocarriers with programmable linkage. The utility of highly optimized combinations of TOs, targeting ligands and nanocarriers enables therapeutic molecules to reach previously inaccessible target tissues/cellular compartments. From the current challenges and emerging trends in delivery of TOs in nanoscale, two appealing directions may hold great potential in promoting the clinical transitions of TOs.

**Table 3:** Advantages and disadvantages of different types of nanocarriers in the TOs delivery.

Nanocarriers	Advantages	Disadvantages
Lipid-based nanoparticles	● Low cytotoxicity ●Long long-circulating and high bioavailability ●Diversity and scale-up feasibility ●High transfection activity	●May trigger immunogenicity ●Difficult to obtain smaller size
Polymeric nanoparticles	●Versatility of structural design ●Stimuli-responsive drug delivery ●Biodegradable ●Controlled drug release	●Difficult to scale-up ●Low drug loading efficiency ●Stability may be a problem for micellar structures
Gold nanoparticles	●Molecular sensing feasibility based on the localized surface plasmon resonance ●Tunability of size and shape ●Photothermal effect ●Easy surface functionalization	●Non-degradable ●Slow body clearance ●Nanotoxicity
Porous nanomaterials	●Structure homogeneity with large active surface area ●Controllable porosity ●High drug-loading capacity ●Capacity to obtain gated supports for on‐demand TOs delivery	●Stability of dispersion requires careful design of surface chemistry ●Possible nanotoxicity for MOF-based materials
DNA/RNA nanoassembly	●Programmability and predictability in TOs loading ●TOs themselves are also building blocks ●Precise structures with addressable modification sites	●High expenses ●Nuclease degradation
Extracellular Vesicles	●Good biocompatibility and low immunogenicity ●Tumor-homing ability and capacity to cross biological barriers ●Superior delivery efficiency ●Tunable surface receptors for site-specific delivery	●No standard methodologies ●Heterogeneous vesicle structures

***Smart Delivery of TOs by Stimuli-responsive Nanocarriers.*** The unique properties of tumor microenvironment (for instance, acid environment, redox state, hypoxia, and specific enzymatic activity) offer a variety of stimulus to realize more complex ‘smart’ delivery. Therefore, what the important aspect of the stimuli-responsive solutions is that the TOs functions in particular action site. To further improve the efficiency of treatment, enzymes/acid-activate ligands and endogenous or exogenous stimulus-responsive nanostructure can be used to release captured nanoparticles to the tumor site. Tumor targeting ligands appear on unshielded nanoparticles, and redirecting particles into the tumor through secondary targeting could be the solution to this challenge. Several stimuli-responsive nanomedicines are yet remaining at the clinical phase, such as Opaxia [412], ThermoDox [413], and AuroShell [414]. Until now, the only responsive nanomedicine, Visudyne, was approved by the FDA utilized in photodynamic therapy. Obviously, many approved drug formulations have been fully evaluated and adequately improved while an important feature of these clinical nanocarriers is their simple formulation. Therefore, as for the majority of stimuli-responsive nanocarriers for TOs delivery that were of sophisticated assemblies and compositional deliberations, the balance between these smart TOs delivery systems and scaling up to the manufacturing scale is the key to prompt the clinical application of responsive nanomedicines.

***Combination of Therapies of TOs with Other Emerging Treatments.*** Combination therapies involve two or more treatments targeting cancer-associated signaling or pathways, and is generally better than the mono-therapy approach in combating cancer [415], considering the multiple mechanisms of cancer occurrence, development, and drug resistance. It has been proved that the combination therapy with in a synergistic manner can decrease the therapeutic dosage of each individual drug (or treatment) and reverse multi-drug resistance [416]. Recent advances in a variety of multifunctional nanocarriers is expected to extend the success of traditional combination therapy to a novel class of therapeutic categories, like chemo-gene [417], photo-gene [418], and immune-gene therapy [419,420]. These strategies have greatly enhanced the effectiveness of single gene therapy and are beneficial to accelerate the clinical transformation of anticancer TOs to a higher extent.

## CRediT authorship contribution statement

**Lei Wu:** Writings and revisions of “Introduction”, “4.2 Polymeric nanoparticles”, “4.3 Gold nanoparticles”, “4.4 Porous nanomaterials”, “4.5 DNA/RNA nanoassembly”, “4.7 Imaging-guided drug delivery systems (IGDD)”, and “4.8 Tumor microenvironment responsive TOs delivery”; Organization of Table 3. **Wenhui Zhou:** Writings of “2 Classifications of the therapeutic oligonucleotides”, “4.6 Extracellular Vesicles”, and “5 Nanocarrier based anticancer oligonucleotide in clinic” sections; Table 1 and Table 2 organizations; Copyright applications for all cited Figures. **Lihua Lin:** Writings and revisions of “4.1 Lipid-based nanoparticles” section. **Anhong Chen:** Writing of “3 Obstacles that prevent the clinical transformation of TOs for cancer therapy” section. **Jing Feng:** Writings polishing. **Xiangmeng Qu:** Supervision, Writing – review & editing, of whole manuscript, focusing on the manuscript organizations for different sections. **Hongbo Zhang:** Supervision, Writing – review & editing, of whole manuscript, focusing on manuscript organizations and aspects related to the clinical translations. **Jun Yue:** Supervision, Writing – review & editing, of whole manuscript, focusing on key issues related to different nanoparticle formulations; Writings of “Abstract” and “6 Conclusions and Future Perspectives” sections.

## Declaration of competing interest

The authors declare that they have no known competing financial interests or personal relationships that could have appeared to influence the work reported in this paper.
